# Multiscale characterization of cortical signatures in positive and negative schizotypy: a worldwide ENIGMA study

**DOI:** 10.1038/s41380-026-03547-x

**Published:** 2026-04-18

**Authors:** Matthias Kirschner, Benazir Hodzic-Santor, Leda Kennedy, Justine Y. Hansen, Mathilde Antoniades, Igor Nenadić, Tilo Kircher, Axel Krug, Tina Meller, Udo Dannlowski, Dominik Grotegerd, Kira Flinkenflügel, Susanne Meinert, Tiana Borgers, Janik Goltermann, Tim Hahn, Joscha Böhnlein, Elisabeth J. Leehr, Carlotta Barkhau, Alex Fornito, Aurina Arnatkeviciute, Mark A. Bellgrove, Jeggan Tiego, Pamela DeRosse, Melissa Green, Yann Quidé, Christos Pantelis, Raymond C. K. Chan, Yi Wang, Ulrich Ettinger, Martin Debbané, Melodie Derome, Christian Gaser, Bianca Besteher, Kelly Diederen, Tom J. Spencer, Josselin Houenou, Edith Pomarol-Clotet, Raymond Salvador, Wulf Rössler, Lukasz Smigielski, Veena Kumari, Preethi Premkumar, Haeme R. P. Park, Kristina Wiebels, Imke Jansen, James Gilleen, Paul Allen, Jan-Bernard Marsman, Irina Lebedeva, Alexander Tomyshev, Anne-Kathrin Fett, Iris Sommer, Sanne Koops, Phillip Grant, Asia Ferrari, Bin Wan, Indrit Bègue, Dennis Hernaus, Maria Jalbrzikowski, Casey Paquola, Sara Larivière, Boris Bernhardt, Sofie L. Valk, Bratislav Misic, Theo G. M. van Erp, Jessica A. Turner, Paul M. Thompson, Andre Aleman, Alain Dagher, Stefan Kaiser, Gemma Modinos

**Affiliations:** 1 Department of Psychiatry, University Hospitals of Geneva, Geneva, Switzerland; 2 McConnell Brain Imaging Centre, Montreal Neurological Institute, McGill University, Montreal, QC Canada; 3 Synapsy Center for Neuroscience and Mental Health Research, University of Geneva, Geneva, Switzerland; 4 Psychiatric University Hospital Zurich, University of Zurich, Zurich, Switzerland; 5 Department of Psychiatry, University of California San Diego, San Diego, CA USA; 6 Children’s Hospital of Philadelphia, University of Pennsylvania, Philadelphia, USA; 7 Center for Biomedical Image Computing and Analytics, Perelman School of Medicine, University of Pennsylvania, Philadelphia, PA USA; 8 University of Marburg, Marburg, Germany; 9 Department of Psychiatry and Psychotherapy, University Hospital Bonn, Bonn, Germany; 10 Institute for Translational Psychiatry, University of Münster, Münster, Germany; 11 Department of Psychiatry, Medical School and University Medical Center OWL, Protestant Hospital of the Bethel Foundation, Bielefeld University, Bielefeld, Germany; 12 German Center for Mental Health (DZPG), Site Jena Magdeburg Halle, Jena, Germany; 13 Center for Intervention and Research on Adaptive and Maladaptive Brain Circuits Underlying Mental Health (C-I-R-C), Site Jena Magdeburg Halle, Jena, Germany; 14 Institute for Translational Neuroscience, University of Münster, Münster, Germany; 15 Department of Psychiatry and Psychotherapy, Charité University Medicine, Berlin, Germany; 16 Turner Institute for Brain and Mental Health, School of Psychological Sciences and Monash Biomedical Imaging, Monash University, Melbourne, VIC Australia; 17 Stony Brook University, Stony Brook, NY USA; 18 Division of Psychiatry Research, Zucker Hillside Hospital, Northwell Health, Glen Oaks, NY USA; 19 The Feinstein Institutes for Medical Research, Center for Psychiatric Neuroscience, Manhasset, NY USA; 20 Department of Psychiatry, Donald and Barbara Zucker School of Medicine at Hofstra/Northwell, Hempstead, NY USA; 21 School of Clinical Medicine, University of New South Wales (UNSW), Sydney, NSW Australia; 22 School of Psychology, The University of New South Wales (UNSW), Sydney, NSW Australia; 23 Neuroscience Research Australia (NeuRA), Randwick, NSW Australia; 24 Department of Psychiatry, University of Melbourne, Carlton South, VIC Australia; 25 State Key Laboratory of Cognitive Science and Mental Health, Institute of Psychology, Chinese Academy of Sciences, Beijing, China; 26 University of Bonn, Bonn, Germany; 27 Faculty of Psychology and Educational Sciences, University of Geneva, Geneva, Switzerland; 28 Research Department of Clinical, Educational and Health Psychology, University College London, London, UK; 29 Department of Psychiatry and Psychotherapy, Jena University Hospital, Jena, Germany; 30 Department of Neurology, Jena University Hospital, Jena, Germany; 31 Department of Psychosis Studies, King’s College London, London, UK; 32 Department of Psychiatry, University of Melbourne and Mental Services, Northern Health, Victoria, Australia; 33 FIDMAG Germanes Hospitalàries Research Foundation & CIBERSAM, ISCIII, Barcelona, Spain; 34 Institute of Psychiatry, School of Medicine, University of Sao Paulo, Sao Paulo, Brazil; 35 Brunel University London, Uxbridge, UK; 36 School of Psychology, University of Auckland, Auckland, New Zealand; 37 Vrije Universiteit Amsterdam, Amsterdam, the Netherlands; 38 University of Manchester, Manchester, UK; 39 Neuroimaging, Institute of Psychiatry, Psychology, Neuroscience, King’s College London, London, UK; 40 Department of Biomedical Sciences of Cells and Systems, University Medical Center Groningen, University of Groningen, Groningen, the Netherlands; 41 Mental Health Research Center, Moscow, Russian Federation; 42 Department of Psychology and Neuroscience, City St George’s, University of London, School of Health and Medical Sciences, London, UK; 43 Fresenius University of Applied Sciences, Frankfurt am Main, Germany; 44 Department of Psychiatry & Neuropsychology, Mental Health and Neuroscience (MHeNs) Research Institute, Maastricht University, Maastricht, Netherlands; 45 Department of Psychiatry and Behavioral Sciences, Boston Children’s Hospital, Boston, MA USA; 46 Department of Psychiatry, Harvard Medical School, MA Boston, USA; 47 Institute of Neuroscience and Medicine (INM-7: Brain & Behaviour) Research Centre Jülich, Jülich, Germany; 48 Department of Medical Imaging and Radiation Sciences, Université de Sherbrooke, Sherbrooke, QC Canada; 49 Max Planck Institute for Human Cognitive and Brain Sciences, Leipzig, Germany; 50 Institute of Systems Neuroscience, Heinrich Heine University Düsseldorf, Düsseldorf, Germany; 51 Clinical Translational Neuroscience Laboratory, Department of Psychiatry and Human Behavior, University of California Irvine, Irvine, CA USA; 52 Center for the Neurobiology of Learning and Memory, University of California Irvine, Irvine, CA USA; 53 Department of Psychiatry and Behavioral Health, the Ohio State University, Columbus, OH USA; 54 Imaging Genetics Center, Mark and Mary Stevens Neuroimaging & Informatics Institute, Keck School of Medicine of the University of Southern California, Marina del Rey, CA USA; 55 Department of Neuropsychology and Psychopharmacology, Faculty of Psychology and Neuroscience, Maastricht University, Maastricht, the Netherlands; 56 Department of Psychological Medicine, Institute of Psychiatry, Psychology, and Neuroscience, King’s College London, London, UK; 57 MRC Centre for Neurodevelopmental Disorders, King’s College London, London, UK

**Keywords:** Schizophrenia, Neuroscience, Psychology, Psychiatric disorders

## Abstract

Positive and negative schizotypy reflect distinct patterns of subclinical traits in the general population associated with neurodevelopmental and schizophrenia-spectrum pathologies. Yet, a comprehensive characterization of the unique and shared neuroanatomical signatures of these schizotypy dimensions is lacking. Leveraging 3D brain MRI data from 2730 unmedicated healthy individuals, we identified neuroanatomical profiles of positive and negative schizotypy and systematically compared them with disorder-specific, microarchitectural, neurotransmitter-level, and connectome measures. Positive and negative schizotypy were associated with distinct cortical signatures, of predominantly thinner frontal and thicker paralimbic cortical areas, respectively. These cortical signatures of positive and negative schizotypy were differentially linked to brain-wide cortical patterns of schizophrenia-spectrum (clinical high-risk for psychosis, schizophrenia) and neurodevelopmental conditions (ADHD, autism spectrum disorder and 22q11.2 deletion syndrome). Additionally, the positive and negative schizotypy-related cortical profiles mapped onto different local attributes of gene expression, cortical myelination, D1, and histamine receptor distributions. Network models further showed that positive and negative schizotypy cortical signatures were spatially associated with cortical hubs, suggesting that highly interconnected regions are more vulnerable to the morphological differences associated with both schizotypy dimensions. Finally, predominantly sensorimotor-to-association and paralimbic areas emerged as epicenters with connectivity profiles significantly linked to the schizotypy-related cortical patterns. Collectively, this study identified cortical signatures of positive and negative schizotypy traits that are embedded along multiple scales of cortical organization and neuropsychiatric pathologies. Our work yields novel insights into how neurobiology and brain architecture may guide neuroanatomical vulnerability and resilience to psychopathology in the general population.

## Introduction

Schizophrenia and related psychotic disorders exist on a spectrum, and the associated symptoms vary along a continuum from health to illness [1–4]. Schizotypy describes a set of multidimensional traits in the general population that are associated with genetic, neurobiological, and behavioral liability to psychotic and related disorders [1, 5, 6]. Positive and negative schizotypy represent subclinical expressions of broader dimensions of psychopathology that mirror the positive and negative symptom domains observed in schizophrenia [4, 7, 8]. The positive dimension (or psychoticism) is characterized by unusual perceptual experiences, suspiciousness, and reality distortion; and the negative dimension (or detachment) encompasses introversion (low extraversion), social withdrawal, and diminished motivation. Both positive and negative schizotypy are associated with the schizophrenia spectrum [8] and also intersect with neurodevelopmental disorders including autism spectrum disorder (ASD), attention-deficit/hyperactivity disorder (ADHD), and 22q11.2 deletion syndrome (22q11DS). Individuals with ASD, ADHD, and 22q11DS tend to have high levels of schizotypal traits [9–13], and ADHD in childhood/adolescence is predictive of higher schizotypal traits in adulthood [14]. In addition, more specific associations between negative schizotypy and ASD [11, 13, 15] and positive schizotypy and 22q11DS [16, 17] have been reported. Taken together, these findings suggest common but also divergent associations of positive and negative schizotypy with schizophrenia spectrum disorders (SSD) as well as neurodevelopmental conditions. Thus, characterizing the brain’s morphometric signatures of positive and negative schizotypy may inform both neurobiological pathways and protective mechanisms related to schizophrenia-spectrum and neurodevelopmental conditions, without confounding by antipsychotic medications or comorbidities with other mental health disorders.

While subtle structural and functional alterations have been associated with schizotypy and subclinical psychotic symptoms in the general population [18–29], methodological heterogeneity and small sample sizes in individual studies have limited previous research [30]. Recognizing these challenges, the ENIGMA Schizotypy Working Group recently coordinated an international analysis of morphometric profiles associated with general schizotypy (n = 3004 individuals), using standardized image analysis, quality assurance and statistical analysis pipelines across cohorts [31]. We identified a distinct neuroanatomical profile characterized by thicker medial orbitofrontal/ventromedial prefrontal cortex (mOFC/vmPFC) associated with higher total schizotypy scores [31]. Furthermore, brain-wide cortical thickness patterns in schizotypy [31] were significantly associated with cortical thinning observed in schizophrenia [32], suggesting a neurobiological continuum within the extended psychosis phenotype. Parallel research has extended this perspective, showing that genetic risk, clinical high-risk and neuropsychiatric conditions, including schizophrenia, share spatial similarities in cortical alterations [33–35]. These shared patterns may be driven by a complex interplay of molecular and connectomic vulnerabilities [36] as well as neurotransmitter profiles [37], that manifest as coordinated alterations in cortical thickness [38, 39]. In this context, two network mechanisms are proposed to guide such coordinated processes of morphometric alterations across risk stages and pathologies. First, it has been demonstrated that both structural and functional cortical hubs are more susceptible to alterations in neuropsychiatric conditions [36, 38, 40–42]. Second, it has been shown that disease-specific and cross-disorder alteration patterns are constrained by the normative connectivity profiles of distinct brain regions. This suggests that cortical alterations propagate in a network-like fashion from one or more epicenters to the most closely connected brain regions [36, 38, 40, 42–46]. However, the extent to which these mechanisms occur on a continuum and may reflect subtle neuroanatomical variations associated with schizotypal traits in the general population remains unknown.

Here we address these questions by conducting a multiscale analysis of the neuroanatomical variations associated with positive and negative schizotypy. We first provide a large-scale meta-analysis of neuroanatomical signatures of positive and negative schizotypy, drawing on comprehensive data of 2730 unmedicated healthy individuals from the ENIGMA-Schizotypy working group. We then systematically compare the cortical thickness profiles associated with positive and negative schizotypy to several existing resources of brain data spanning disease-related cortical abnormality maps, micro-architecture, and global connectomics [36, 37]. Specifically, we contrast the cortical alteration patterns of positive and negative schizotypy with meta-analytic cortical alteration maps of schizophrenia spectrum conditions, including those found in groups of people at clinical high-risk for psychosis [47] and with established schizophrenia [32]. To uncover common and distinct associations with neurodevelopmental disorders, we also compare the schizotypy-related cortical patterns with cortical effect size maps of ASD [48], ADHD [49], and 22q11DS [50]. In addition, we compare the cortical profiles of positive and negative schizotypy to detailed brain maps of multiple microarchitectural measures, including gene expression, metabolism, and myelination, as well as global connectomic measures such as number of connections, centrality, and diversity, and delineate the role of specific neurotransmitters. To further examine the association between schizotypal cortical maps and normative brain architecture, two network susceptibility models are applied: hub vulnerability, which compares regional network centrality with the magnitude of disease-specific atrophy; and epicenter mapping, which identifies regions whose typical connectivity profiles most closely reflect the spatial pattern of disease-specific alterations. By focusing on the relative spatial organization of effect sizes, these multiscale analyses allow subtle but coherent cortical variations associated with schizotypy to reveal meaningful links to molecular, developmental, and network-level features. Overall, we uncover how positive and negative schizotypy map onto multiple scales of biological and cortical organization, offering new insights into the neurobiological basis of these complex schizophrenia-related traits in the general population.

## Results

We studied 2730 unmedicated healthy individuals (12–68 years, 47% male) with varying levels of self-reported positive and negative schizotypy, derived from 31 sites of the worldwide ENIGMA Schizotypy Working Group (Tables S1-4). Using standardized protocols on 3D brain MRI scans, cortical thickness (CT) and surface area (SA) of 68 gray matter brain regions were measured based on the Desikan-Killiany-Tourville (DKT) anatomical atlas [51], and subcortical volume (SV) of 16 subcortical regions using FreeSurfer [52, 53] (http://surfer.nmr.mgh.harvard.edu) and standardized ENIGMA protocols (http://enigma.ini.usc.edu/protocols/imaging-protocols/). Following previous ENIGMA work [31, 32], we examined the associations between regional CT, SA, SV values and either positive or negative schizotypy scores using partial correlations including age and sex (and intracranial volume (ICV) for SV) as covariates for each site separately. We generated separate meta-analytic cortical effect size maps for positive and negative schizotypy including the Pearson’s *r* effect sizes from each site using inverse variance-weighted random effects models that account for different sample sizes across sites. We corrected for multiple comparisons using the false discovery rate (FDR; p_FDR_ < 0.05) [54].

### Morphometric profiles of positive and negative schizotypy

**Fig. 1:**
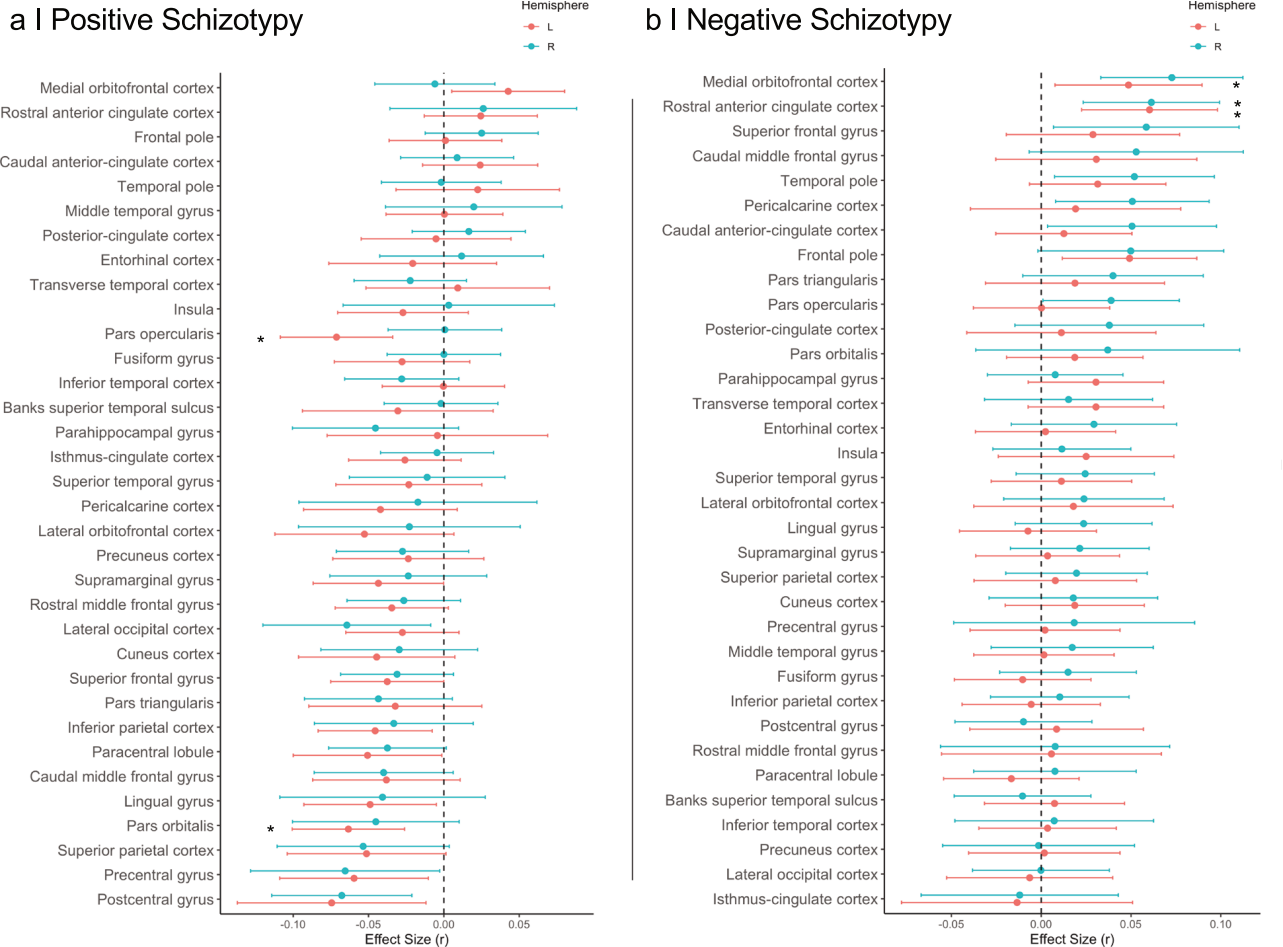
Effect sizes of partial correlation (*r*) between cortical thickness and schizotypy dimensions. Partial correlations (corrected for age and sex) between regional cortical thickness and **(a)** positive schizotypy, and **(b)** negative schizotypy. *Regions with *p*_*FDR*_ < 0.05.

**Fig. 2:**
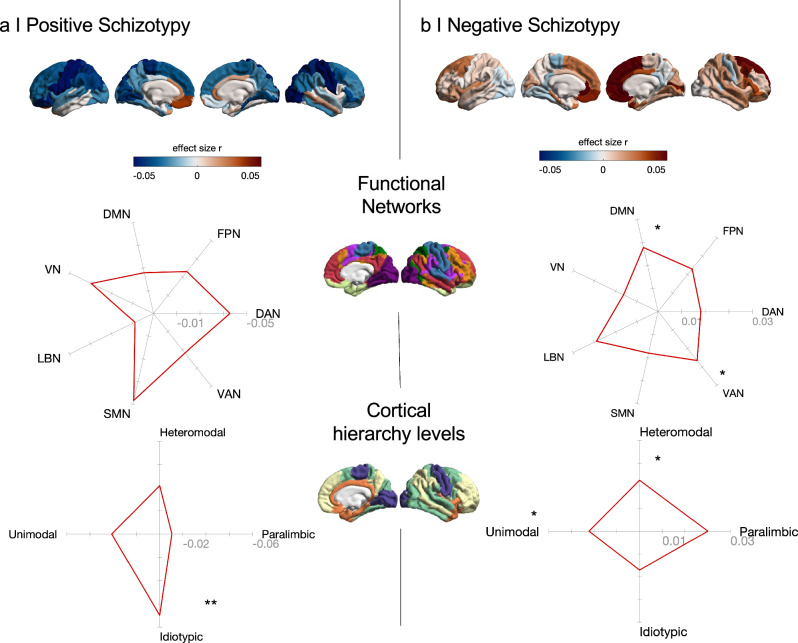
Cortical effects in positive and negative schizotypy aggregate within functional networks and cortical hierarchy levels. Cortical surfaces show the mean effect sizes (partial correlation r) for **(a)** positive and **(b)** negative schizotypy. Spider/Radar plots stratify the mean effect size values for positive and negative schizotypy according to functional networks [55] and cortical hierarchy levels [56]. Significance was assessed with a one sample *t*-test against 10000 null models using spin permutation testing to account for spatial autocorrelation. In positive schizotypy, idiotypic-type regions exhibit significantly lower *t* statistics relative to spin permutation null models. In negative schizotypy, VAN, DMN functional networks, as well as heteromodal-, and unimodal-type regions show significantly higher t statistics relative to spin permutation models. DAN, Dorsal Attention Network (green); DMN, Default Mode Network (red); FPN, Fronto-Parietal Network (orange); LBN, Limbic Network (light green); SMN, Somato-Sensory Network (blue); VAN, Ventral Attention Network (pink); VN, Visual Network (purple). Cortical hierarchy levels: Idiotypic (purple-blue); Unimodal (green); Heteromodal (yellow); Paralimbic (orange). *<0.1 FDR-p_spin_**<0.05 FDR-p_spin_.

When assessing the relationship between both schizotypy dimensions and regional CT, we found that higher positive schizotypy was significantly associated with thinner left inferior frontal gyrus *pars opercularis* (*r* = −0.07, *p*_FDR_ = 0.018, 95% CI [−0.109, −0.034]) and *pars orbitalis* (*r* = −0.06, *p*_FDR_ = 0.043, 95% CI [−0.101, −0.026]), and at trend level with thinner right postcentral gyrus (*r* = −0.07, *p*_FDR_ = 0.08, 95% CI [−0.137, −0.012]) (Figs. 1, 2, Table S5). In contrast, higher negative schizotypy was significantly associated with thicker right mOFC/vmPFC (right, *r* = 0.07, *p*_FDR_ = 0.01, 95% CI [0.033, 0.112]; left, *r* = 0.05, *p*_FDR_ = 0.25, 95% CI [0.008, 0.090]) and bilateral rostral anterior cingulate cortex (right, *r* = 0.06, *p*_FDR_ = 0.03, 95% CI [0.023, 0.099]; left, *r* = 0.06, *p*_FDR_ = 0.03, 95% CI [0.022, 0.098]) (Figs. 1, 2, Table S6). Moderator analyses (Tables S7–S12) indicated that the type of schizotypy questionnaire, FreeSurfer version, and scanner field strength did not significantly influence the specific cortical regions showing significant associations with positive or negative schizotypy (all pFDR > 0.05 for these regions). Although several regions across the cortex showed significant questionnaire-moderation effects (Tables S7, S9), these did not overlap with the regions identified as significantly associated with positive and negative schizotypy. Meta-analysis of SA and SV revealed no significant associations with positive or negative schizotypy, and these measures were not included in further multiscale analyses (see Tables S13-S16 for details). Taken together, these findings suggest opposite patterns of lower regional CT in individuals with higher positive schizotypy and higher regional CT in individuals with higher negative schizotypy (Figs. 1, 2).

### Stratification of cortical thickness profiles of schizotypy to functional networks and cortical organization

To further contextualize the distinct schizotypy-dimension spatial patterning, we stratified the effect size maps of cortical thickness associations with positive and negative schizotypy according to intrinsic functional brain networks [55] and levels of cortical hierarchy [56]. In positive schizotypy, thickness reductions were predominantly observed in the somatomotor (SMN)/visual (VN) and dorsal attention (DAN) networks, but these effects were not significant compared to null models preserving spatial autocorrelation (all *p*_spin_ > 0.05). Regarding cortical hierarchy levels, we observed a significant aggregation of the association between positive schizotypy and lower cortical thickness in idiotypic regions (mean = −0.05, *t* = −183.52, FDR-*p*_spin_ = 0.045). For negative schizotypy, the association with greater cortical thickness showed nominal enrichment across both functional networks and cortical hierarchy levels (*p*_spin_ < 0.05), which did not survive FDR correction. Specifically, negative schizotypy-related associations were stronger than spatial null models in the visual attention network (VAN) (mean=0.019, *t* = 52.28, FDR-*p*_spin_ = 0.055) and the default mode network (DMN) (mean=0.019, *t* = 52.55, FDR-*p*_spin_ = 0.098). With respect to cortical hierarchy levels, negative-schizotypy associations were more pronounced in heteromodal (mean=0.016, *t* = 70.04, FDR-*p*_spin_ = 0.066) and unimodal regions (mean=0.014, *t* = 56.97, FDR-*p*_spin_ = 0.098) (Fig. 2). Taken together, these observations suggest that the spatial patterns of associations between CT and higher positive and negative schizotypy may be concentrated in different functional networks (SMN/VN/DAN vs. DMN/VAN) and levels of cortical organization (idiotypic vs unimodal/heteromodal). However, enrichment effects for negative schizotypy should be interpreted with caution, as they did not meet the strict FDR q = 0.05 threshold and instead fall within a marginal range (0.05 ≤ FDR < 0.10).

### Cortical pattern similarity of schizotypy dimensions with neurodevelopmental disorders

**Fig. 3:**
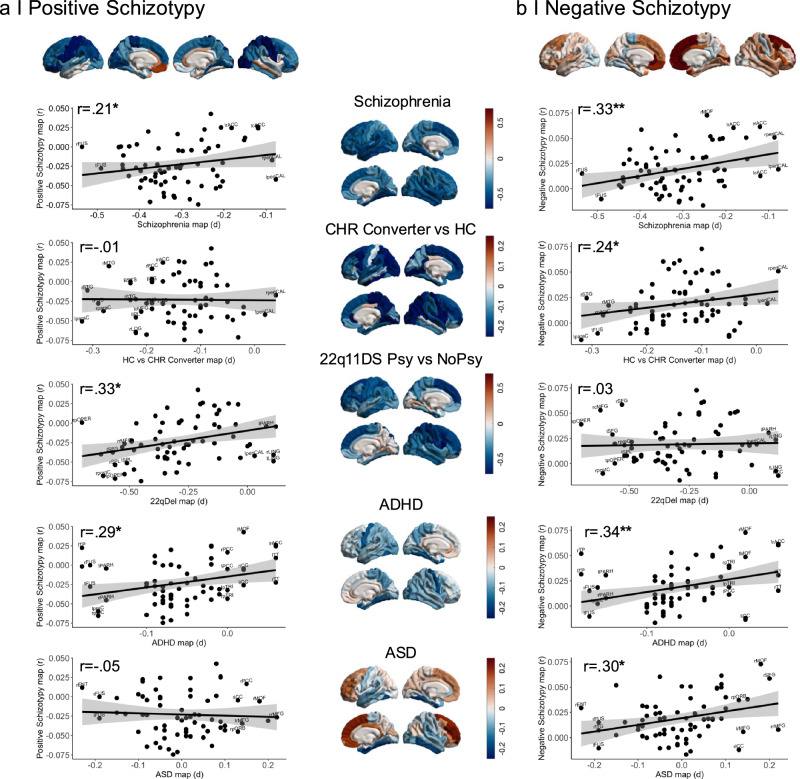
Cortical pattern similarity of schizotypy dimensions with schizophrenia and neurodevelopmental disorders. The cortical surfaces show the partial correlation effect sizes of positive and negative schizotypy, and the case-control effect sizes (Cohen’s D) of schizophrenia, CHR individuals who converted to psychosis, 22q11DS-psychosis vs non-psychosis individuals, children with ADHD (age 4–14 years), and ASD on cortical thickness from ENIGMA meta-/mega-analyses of each disorder [32, 47–50]. Significance was assessed using spin permutation (10000 repetitions) to account for spatial autocorrelation between two brain maps [110]. Scatterplots show the correlation of each disorder map with **(a)** cortical effects of positive schizotypy and **(b)** cortical effects of negative schizotypy. *<0.1 FDR-p_spin_**<0.05 FDR-p_spin_.

We tested whether the spatial patterns of cortical effect size maps of positive and negative schizotypy (partial correlation, *r*, Fig. 2) are related to case-control cortical effect size patterns (Cohen’s *d*) of schizophrenia-spectrum and neurodevelopmental conditions. To this end, we systematically correlated the cortical effect size maps of both schizotypy dimensions with the case-control effect size maps of each disorder derived from the recently published ENIGMA studies [32, 48–50]. Specifically, we included meta- and mega-analytic case-control cortical maps of individuals at clinical high-risk (CHR) for psychosis who subsequently developed psychosis (CHR-converters) [47], schizophrenia [32], 22q11DS individuals with psychosis (22q11DS-psychosis) [50], ADHD [49], and ASD [48]. Both positive and negative schizotypy patterns of cortical differences showed small-to-moderate spatial correlations with the cortical alteration pattern in schizophrenia (positive: *r* = 0.221, FDR-*p*_spin_ = 0.072; negative: *r* = 0.334, FDR-*p*_spin_ = 0.022), with only the association for negative schizotypy surviving FDR correction. Spatial correlations with the cortical thickness pattern of CHR-converters showed a divergent pattern across schizotypy dimensions. The negative schizotypy-related cortical profile showed a spatial positive correlation with the CHR-converter-related cortical thickness pattern (*r* = 0.24, FDR-*p*_spin_ = 0.052), whereas the positive schizotypy-related cortical profile showed essentially no correlation (*r* = −0.001, FDR-*p*_spin_ = 0.617) (Fig. 3). Similar results were found for the cortical profiles of all CHR individuals (including those who did not develop psychosis) compared to healthy controls (Fig. S1).

With respect to neurodevelopmental disorders, the cortical pattern of negative schizotypy showed a significant spatial correlation with the cortical alteration map of ADHD (*r* = 0.34, FDR-*p*_spin_ = 0.020). The correlation with the ASD cortical profile did not survive FDR correction (*r* = 0.30, FDR-*p*_spin_ = 0.068). In contrast, there was minimal spatial correspondence between negative schizotypy and 22q11DS-psychosis (*r* = 0.03, FDR-*p*_spin_ = 0.5) (Fig. 3). For positive schizotypy, spatial correlations were observed with the cortical alteration profiles of 22q11DS-psychosis (*r* = 0.33, FDR-*p*_spin_ = 0.065) and ADHD (*r* = 0.29, FDR-*p*_spin_ = 0.064) although neither survived FDR correction. In contrast, no spatial correspondence was found between the cortical profiles of positive schizotypy and ASD (*r* = −0.05, FDR-*p*_spin_ = 0.617) (Fig. 3). Together, these results suggest that both schizotypy dimensions share spatial correspondence with the cortical alteration pattern established in schizophrenia, although only the association for negative schizotypy survives FDR correction. Furthermore, the results indicate overlapping associations with the cortical alteration pattern of ADHD, and dimension-specific spatial associations with the cortical profiles of early psychosis, 22q11DS-psychosis, and ASD. However, these shared and dimension-specific associations should be interpreted with caution as only the association between negative schizotypy and ADHD remained significant after FDR correction.

### Molecular, connectivity and neurotransmitter contributions to cortical morphology of schizotypy dimensions

**Fig. 4:**
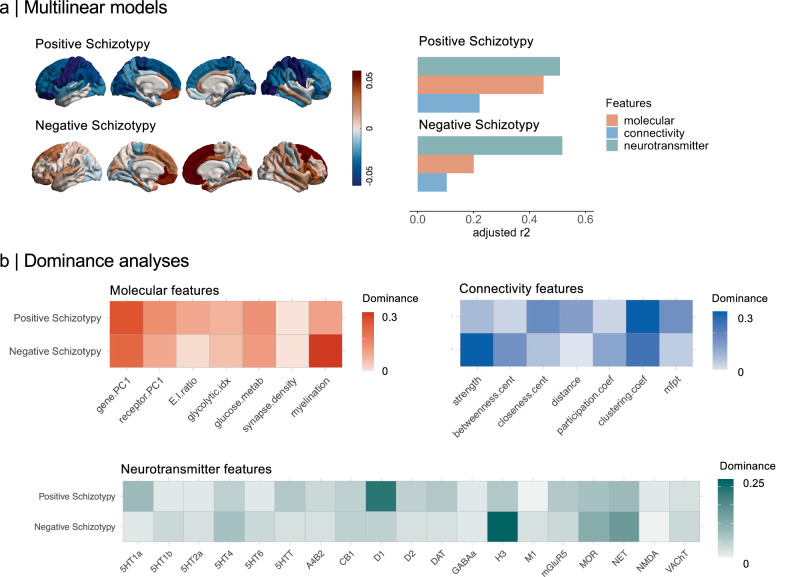
Molecular, connectivity, and neurotransmitter contributions to cortical morphology of schizotypy dimensions. **(a)** In total, six multilinear models were fit between molecular, structural connectivity, and neurotransmitter predictors to cortical morphology maps of positive and negative schizotypy. Adjusted *R2* is shown in the bar plot (*orange*: molecular; *blue*: connectivity; *green*: neurotransmitter). (**b)** Dominance analysis was applied to assess the contribution of each input variable to the fit of the model. This was done separately for the molecular (*orange*), connectivity (*blue*), and neurotransmitter (*green*) predictors. Standardized dominance values are shown (range 0-1) that represent the proportion of explained variance (relative contribution) attributable to each predictor within its multilinear model, with all predictors for a model summing to 1. Molecular predictors: gene PC1 = first component of 11,560 genes’ expression; receptor PC1 = first component of 18 PET-derived receptor/transporter density; E:I ratio = excitatory:inhibitory receptor density ratio; glycolytic index = amount of aerobic glycolysis; glucose metabolism = [^18^F]-labelled fluorodeoxyglucose (FDG) PET image; synapse density = synaptic vesicle glycoprotein 2 A (SV2A)-binding [^11^C]UCB-J PET tracer; myelination = T1w/T2w ratio. Connectivity predictors: strength = sum of weighted connections; betweenness = fraction of all shortest paths traversing region *i*; closeness = mean shortest path length between region *i* and all other regions; Euclidean distance = mean Euclidean distance between region *i* and all other regions; participation coefficient = diversity of connections from region *i* to the seven Yeo-Krienen resting-state networks^46^; clustering = fraction of triangles including region *i*; mean first passage time = average time for a random walker to travel from region i to any other region. Neurotransmitter predictors: Neurotransmitter predictors used in the multilinear models included 18 cortical receptor/transporter maps derived from PET tracer studies. These include dopamine (D1, D2, DAT), norepinephrine (NET), serotonin (5-HT1A, 5-HT1B, 5-HT2A, 5-HT4, 5-HT6, 5-HTT), acetylcholine (α4β2, M1, VAChT), glutamate (mGluR5), GABA (GABA_A_), histamine (H3), cannabinoid (CB1), and opioid (MOR). Data collection and processing of all PET maps into 68 cortical DKT regions is detailed in Hansen et al [37].

We next examined how cortical differences in positive and negative schizotypy map onto specific molecular gradients, structural network properties, and neurotransmitter receptor/transporter distributions. To address these questions, we used brain maps derived from recent publications by Hansen and colleagues [36, 37]. These include molecular, structural connectivity and neurotransmitter profiles that are parcellated according to the same 68 regions of the DKT atlas, allowing for direct comparison with the cortical morphology pattern of positive and negative schizotypy (for details see [36, 37] and Materials and Methods). Following previous work [36, 37], we fitted multiple linear models using three sets of predictors – molecular, structural connectivity, and neurotransmitter features – against cortical morphology maps for positive and negative schizotypy separately, resulting in six different model fits (Fig. 4a). We found that the cortical morphology of positive schizotypy was significantly predicted by each set of features (adj *R²* > 0.2, *p* < 0.005) (Fig. 4a). Cortical morphology of negative schizotypy was significantly predicted by molecular and neurotransmitter features (both adj *R²* > 0.2, *p* < 0.005), while the model fit including connectivity features did not reach significance (adj *R²* = 0.1, *p* = 0.06). These findings suggest that cortical morphological patterns of positive and negative schizotypy are both associated with both molecular and neurotransmitter features.

To further examine the contribution (“dominance”) each input variable has on the cortical morphology patterns of both schizotypy dimensions, we applied dominance analyses to each multilinear model separately (Fig. 4b). Dominance analysis quantifies the relative importance of each predictor in explaining variance in cortical morphology, and we report standardized dominance values (proportion of total explained variance) for each input feature (Fig. 4b). In addition to the results above, dominance analyses revealed that the importance of predictors varied across schizotypy dimensions (Fig. 4b). For the CT pattern related to positive schizotypy, the first principal gene expression gradient from Allen Human Brain Atlas (a potential proxy for cell type distribution [57–60]), and dopamine D1 receptor density were the most dominant predictors. In contrast, for the CT pattern associated with negative schizotypy, myelination and histamine H3 receptor density demonstrated the greatest dominance in the multilinear models (Fig. 4b). Other predictors such as synapse density or NMDA receptor density were consistently less dominant in predicting cortical morphology of both schizotypy dimensions. Collectively, these findings suggest distinct biological mechanisms underlying the cortical morphology profiles of positive and negative schizotypy, with different molecular and neurotransmitter predictors demonstrating variable influence across these dimensions.

### Hub vulnerability and epicenters of schizotypy dimensions

**Fig. 5:**
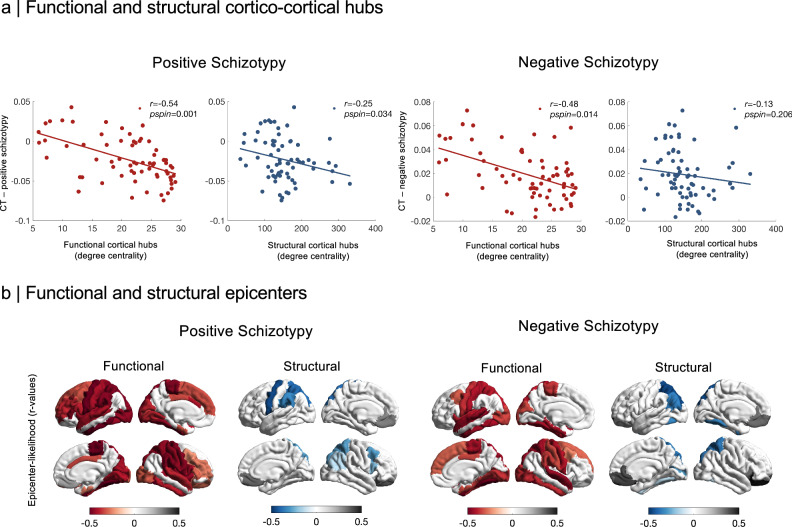
Hub and epicenter models of cortical morphology patterns in positive and negative schizotypy. **(a)** Correlation between cortical morphology patterns of positive and negative schizotypy with normative node-level functional (*red*) and structural (*blue*) degree centrality maps (derived from the healthy adult HCP dataset). In positive schizotypy, regions with high functional or structural centrality are significantly more likely to display reduced cortical thickness and regions with low functional or structural centrality are more likely to display cortical thickness increase. The same association was found for negative schizotypy and functional cortical hub regions but not structural cortical hubs. (**b)** Epicenter maps depicting the strength of associations (correlation coefficients) between the normative region-based functional (*red*) and structural (*blue*) connectivity and cortical morphology maps of positive and negative schizotypy. Significant epicenters with negative *r*-values are regions that are more strongly connected to regions with more reduced cortical thickness in association with positive and negative schizotypy (indicated by red and blue color range respectively). Inversely, significant epicenters with positive r-values are more strongly connected to regions with greater cortical thickness in association with positive and negative schizotypy (indicated by gray color range). Only significant epicenters with *p*_spin_ < 0.05, after spin permutation test (1000 repetitions) are displayed.

We then tested whether the hub vulnerability hypothesis –i.e., that regions with higher network centrality are more susceptible to pathological and maladaptive processes in neuropsychiatric disorders than non-hub regions [40–42, 45, 61]– extends to positive and negative schizotypy. To this end, we examined whether cortical hub regions (defined by higher normative network centrality derived from an independent healthy Human Connectome Project (HCP) sample [62], n = 207) show stronger associations (partial correlation, *r*) with positive or negative schizotypy compared to non-hub regions. We compared the spatial patterns of normative functional and structural node degree centrality (Figs. S2, S3) and the cortical thickness patterns of positive and negative schizotypy (Fig. 2). In positive schizotypy, we found a significant correlation between functional and structural centrality and CT reduction in association with positive schizotypy (functional: *r* = −0.54, *p*_spin_ = 0.003; structural *r* = −0.25, *p*_spin_ = 0.036, Fig. 5a). Conversely, regions with relative CT increase in relation to positive schizotypy showed relatively lower function and structural centrality. In negative schizotypy, the same association was observed for functional but not structural cortical hub regions (functional: *r* = −0.48, *p*_spin_ = 0.018; structural *r* = −0.13, *p*_spin_ = 0.197, Fig. 5a). In other words, cortical hub regions were more likely to display lower CT in relation to either higher positive or higher negative schizotypy scores, whereas regions with lower centrality in the network were more likely to show higher CT in association with either positive or negative schizotypy.

Finally, we aimed to identify putative epicenters of the cortical thickness patterns of positive and negative schizotypy. Epicenters are defined as regions whose normative connectivity profile (derived from the independent healthy adult HCP dataset [62] mentioned above, Figs. S2, S3) most closely resembles disease-related morphological alteration patterns [43, 45, 63, 64]. That is, epicenters are connected to brain regions with the greatest cortical thickness differences. Significant negative epicenter likelihood (R-values) indicates that the connectivity profile of a region is more strongly associated with lower CT (meta-analytic negative partial R-values). A positive epicenter-likelihood indicates that the connectivity profile of a region is more strongly related to greater CT (meta-analytic positive partial R-values). For lower CT associated with positive or negative schizotypy, we observed several functional epicenters distributed across sensorimotor to association regions, and structural epicenters more circumscribed within sensorimotor and parietal cortices (Fig. 5b, Table S17-S18). In contrast, epicenters of greater CT in both positive and negative schizotypy were exclusively localized in the mOFC/vmPFC, lateral OFC, and ACC (Fig. 5b, Table S19-S20). Importantly, significance of all functional and structural epicenter maps remained stable when using complementary network-rewiring null models [44] that conserve degree and distance relationships of the connectome (Supplementary Fig. S4). In summary, epicenters of schizotypy-related CT reduction were more widely distributed across sensorimotor-associative regions, and epicenters of schizotypy-related CT increase were more circumscribed in the mOFC/vmPFC and adjacent regions.

## Discussion

Capitalizing on large-scale neuroimaging data from the ENIGMA Schizotypy Working Group, the key finding of our study is the identification of overlapping and distinct neuroanatomical signatures of positive and negative self-reported schizotypy in the general population. Specifically, higher positive schizotypy scores were associated with a cortical profile of predominantly CT reduction, with the strongest associations in the inferior frontal gyrus. Higher negative schizotypy was associated with a pattern of mostly CT increase, with strongest associations in the mOFC/vmPFC and rostral ACC. The spatial pattern of both CT profiles was constrained to different functional networks and regions with distinct levels of cortical hierarchy. Associations between CT and positive schizotypy were mainly found in idiotypic regions, whereas significant associations with negative schizotypy were mainly localized in the VAN and DMN as well as in heteromodal and unimodal regions. We further observed that the cortical profiles of both schizotypy dimensions were spatially correlated with those in schizophrenia and ADHD. In addition, distinct associations emerged, with positive schizotypy-related cortical alterations correlating with those in 22q11DS-psychosis, and negative schizotypy-related cortical alterations correlating with those in CHR-converters and ASD. These findings suggest overlapping but also divergent neurodevelopmental mechanisms related to positive and negative schizotypy. Linking the cortical profiles of both schizotypy dimensions to microarchitectural measures, distinct molecular and neurotransmitter features emerged as predictors of cortical variation in schizotypy, suggesting specific biological mechanisms involved. Finally, comparing the cortical profiles of both schizotypy dimensions with normative functional and structural connectivity, we found that cortical hubs were more vulnerable to the CT associations with positive and negative schizotypy. In addition, sensori-motor and prefrontal areas emerged as putative epicenters of CT reduction and increase, respectively. Collectively, this work advances our understanding of the neuroanatomy of positive and negative schizotypy in healthy individuals, without confounding by medication or comorbidities, reveals links to schizophrenia-spectrum and related neurodevelopmental conditions, and uncovers relationships with cortical organization, network properties, and biological mechanisms.

Our analyses revealed that higher positive schizotypy predominantly correlated with lower CT, whereas higher negative schizotypy predominantly correlated with higher CT. Interestingly, greater OFC/vmPFC thickness emerged as a signature common to both dimensions, albeit only to a significant degree in negative schizotypy, mirroring our prior meta-analytic results using total schizotypy scores [31]. These findings suggest that neuroanatomical variations observed in positive and negative schizotypy may reflect different neurodevelopmental mechanisms of accelerated versus delayed cortical maturation. Consistent with this notion, the positive and negative schizotypy-related cortical profiles spatially overlapped with those of several psychiatric and neurodevelopmental conditions, including schizophrenia-spectrum conditions, ADHD, 22q11DS, and ASD. We found that the cortical alteration patterns of both schizotypy dimensions were spatially related to those seen in schizophrenia, confirming previous findings of a neuroanatomical continuum across the extended psychosis phenotype [31, 47, 50]. Furthermore, the cortical profile of negative schizotypy, but not positive schizotypy, was spatially related to the cortical alterations seen in CHR individuals who subsequently transitioned to psychosis [47]. Psychometric studies have consistently shown that both positive and negative schizotypy predict schizophrenia-spectrum psychopathology and disorders [1, 5, 65]. Our findings expand this body of work by suggesting that while behaviourally both schizotypy dimensions have been associated with transition to psychosis in CHR individuals [1, 5, 65], neuroanatomically the negative schizotypy dimension profile associates more strongly with the CHR-converter profile. It should be noted that despite the observed spatial associations, the distributed increased CT in negative schizotypy contrasts with the predominantly reduced CT patterns of schizophrenia and CHR converters. This increased CT in negative schizotypy may reflect pre-existing processes prior to the accelerated cortical thinning around the onset of psychosis. Alternatively, the observed divergent pattern - higher CT in negative schizotypy and lower CT in SSD - may indicate the absence of pathological or the presence of compensatory mechanisms in healthy individuals with negative schizotypy. In this context, advances in translating meta-analytic findings into individual vulnerability scores [66–68] could be used to assess longitudinally whether greater similarity to the cortical profile of negative schizotypy improves the prediction of psychosis in CHR individuals.

The coordinated processes of cortical maturation during childhood and adolescence not only reflect typical development but also highlight potential vulnerabilities to neurodevelopmental conditions [35, 69, 70]. Comparing the CT profiles of neurodevelopmental conditions with those of both schizotypy dimensions, we observed overlapping associations of both schizotypy dimensions with ADHD. In addition, unique associations emerged between positive schizotypy and 22q11DS-psychosis, and between negative schizotypy and ASD. These findings are consistent with psychometric studies showing common but also distinct relationships of positive and negative schizotypy with subclinical and clinical symptoms of neurodevelopmental conditions [9, 10, 14–17]. A diagnosis of ADHD during adolescence has been found to predict higher total and negative schizotypy later in life [14], and associations between higher ADHD traits and positive schizotypy have been observed in relatives of individuals with schizophrenia [71, 72] and in healthy individuals [73, 74]. Studies of 22q11DS individuals have consistently reported elevated positive schizotypy scores [9, 16, 75], reflecting the well-established increased risk of developing schizophrenia in this population [76]. In addition, 22q11DS individuals who later developed psychosis exhibited neuroanatomical alterations similar to those seen in schizophrenia [50]. For ASD, diametric associations of higher negative schizotypy with higher autistic traits and an inverse relationship between positive schizotypy and autistic traits have been previously observed [11]. This supports our findings of a specific positive correlation between the CT profiles of negative schizotypy and ASD. Collectively, these findings suggest that schizotypal traits are related to complex variations of cortical development that may manifest in different psychotic and neurodevelopmental psychopathologies.

In this regard, a growing literature on cross-disorder effects has revealed common vulnerabilities affecting multiple scales of cortical organization including transcriptomics, neurotransmitter systems, and microstructural features [34, 36, 38, 77]. Local molecular features of neuroreceptor density, gene expression, and microstructure were found to represent important predictors of disease-specific and cross-disorder patterns of cortical alteration [34, 36]. Our findings show that the cortical profiles of positive and negative schizotypy are not randomly distributed but can be explained by regional variations in molecular attributes and specific neurotransmitters. The cortical profile of positive schizotypy was significantly explained by a regional distribution of gene expression, whereas the cortical morphology associated with negative schizotypy was significantly predicted by differences in T1w/T2w ratios, an MRI proxy for intracortical myelin content. Both gradients of gene expression and cortical myelination follow a sensorimotor to transmodal axis of cortical organization [57, 60, 69, 78, 79] that has been linked to common neuroanatomical effects across neurodevelopmental and psychiatric disorders [34, 36, 69]. Here, we found that cortical profiles of positive and negative schizotypy relate to similar principles of cortical hierarchies suggesting common vulnerability during cortical development. With respect to neurotransmitter systems, previous work found that brain-wide receptor distributions were associated with cortical maps of major psychiatric disorders but not with the cortical profile of overall schizotypy [37]. In the present study, however, decomposing total schizotypy into positive and negative schizotypy revealed significant associations with two different neurotransmitter systems. The cortical differences seen in positive schizotypy were explained by the distribution of cortical D1 receptor density, mirroring the above-mentioned dorsolateral to ventromedial cortical hierarchy [37]. In contrast, the cortical differences associated with negative schizotypy were predicted by the distribution of cortical H3 receptor, reflecting their high density in frontal regions, including the ACC and vmPFC. Cortical histamine modulates multiple cognitive processes [80, 81] and altered H3 receptor function is increasingly recognized as a potential mechanism underlying cognitive impairments in schizophrenia [82, 83]. Therefore, although speculative, the associations of negative schizotypy-related cortical differences and H3 receptor distribution may reflect either compensatory mechanisms that preserve cognitive function or pre-existing factors underlying cognitive deficits in schizophrenia. Future work is needed to further decipher the putative pathological or compensatory nature of these newly identified spatial associations. Collectively, our findings suggest that the cortical patterns of both positive and negative schizotypy follow unifying principles of cortical transcriptomic and microstructural differentiation with additional contributions from distinct neurotransmitter systems. This may embed a common vulnerability to neurodevelopmental and neuropsychiatric conditions, but may also suggest resilience mechanisms. The differential contribution of gene expression and myelination may be further explained by subtle local variations in the cortical profiles of positive and negative schizotypy.

Finally, we contextualized the contribution of normative brain network architecture to the spatial pattern of cortical differences in positive and negative schizotypy. We provide evidence that regions with higher network centrality are more susceptible to cortical differences associated with both schizotypy dimensions. Within the topology of brain networks, centrally located neuronal populations are particularly vulnerable to pathophysiological perturbations due to their higher neuronal activity and concomitant metabolic demands [41, 42, 84]. In addition, the most central hub regions are concentrated in heteromodal association cortices that, due to their prolonged window of developmental plasticity, have been shown to be especially vulnerable to insults during cortical development [69]. Such increased vulnerability of cortical hub regions to pathological processes has been consistently found in neurodegenerative disorders [85–88], and more recently in neurodevelopmental and psychiatric conditions [40, 41, 89]. Several biological mechanisms could lead to a marked susceptibility of hub regions. Extending this literature, our findings demonstrate that hub vulnerability may already manifest itself in cortical variations associated with vulnerability traits such as schizotypy.

Spatial patterns of disease-specific and cross-disorder alterations can be explained, to some extent, by connectivity profiles of specific regions - or epicenters - that most closely resemble the corresponding morphometric alteration profile [36, 38, 40]. Pathological processes in brain disorders may spread in a network-like fashion along the underlying normative connectome architecture [42, 46]. For both positive and negative schizotypy, we observed epicenters of CT reduction in sensorimotor-to-association areas and epicenters of CT increase in the vmPFC/mOFC and ACC. The distributed sensorimotor-to association epicenters of CT reduction align well with recently reported epicenters of cross-disorder cortical abnormalities and co-alteration of neurodevelopmental, neurological and psychiatric disorders [36, 38]. Complementarily, the circumscribed paralimbic epicenters of CT increase closely mirror recent findings across different stages of schizophrenia [40, 43, 44]. Together, these associations with the connectome architecture may reflect how schizotypy-related cortical processes could contribute to both neurodevelopment in general and specific neuroanatomical processes related to psychosis.

The present work has several methodological limitations. First, significant associations of positive and negative schizotypy were only observed with regional CT and even larger sample sizes may be needed to identify associations with subcortical volumes. Although only a small number of regions showed significant associations after FDR correction, this pattern is consistent with prior large-scale studies of subclinical traits in healthy individuals, where effect sizes are typically subtle. The magnitude of our associations is comparable to effects reported for personality traits [90], psychopathology [91], and polygenic risk [35, 92, 93] in the general population, as well as other ENIGMA studies in neurodevelopmental conditions such as ADHD [49] and ASD [48]. Importantly, recent multiscale studies consistently show that subtle but spatially coherent cortical effect-size maps can still carry meaningful biological information, aligning with normative developmental gradients [69] and molecular, transcriptomic, and connectomic features [34, 36, 38]. While such whole-cortex effect-size patterns provide insight beyond single-region significance, an important open question for future research is whether quantitative thresholds, such as minimum regional effect sizes or spatial continuity, are needed to identify robust spatial-pattern inference. Addressing this will require even larger, longitudinal, and multimodal cohorts to test how such distributed patterns emerge and to determine under what conditions subtle brain-wide effects should be leveraged for mechanistic interpretation. Second, the molecular, and neurotransmitter maps are coming from different individuals/datasets, are all derived from healthy individuals, and are only available for cortical regions. Third, the present work considered neuroanatomical signatures of the positive and negative schizotypy based on the large body of research for these dimensions and the available data across sites. However, future efforts of the ENIGMA Schizotypy Working Group will aim to pool the necessary data for the disorganized schizotypy dimension [94]. Fourth, atlas choice represents an additional methodological consideration, as structural network properties can vary across parcellations [95]. We used the DKT atlas because it is the standard within the ENIGMA consortium and provides harmonized cortical thickness measures across 31 international sites, ensuring direct spatial correspondence between all schizotypy-related and case–control maps. Future large-scale multisite neuroimaging initiatives in which raw data can be centrally processed or harmonized across multiple parcellations will be essential for systematically comparing network-level and multiscale findings across atlas resolutions. Finally, the current findings are based on comparisons of meta-analytic cortical profiles of schizotypy dimensions with cortical effect size maps from disorder and at-risk populations, as well as group-level brain maps of molecular, connectivity, and neurotransmitter properties derived from separate samples of healthy participants. As such, these findings do not capture individual variability in multiscale differentiation, nor do they allow for direct comparisons of different populations across the lifespan. However, it should be noted that large-scale multiscale investigations using multiple PET scans and histology at the individual level are not feasible and future work will continue to rely on data aggregation from different resources and cohorts. In addition, these are powerful hypothesis-generating approaches that offer candidate targets for direct experimentation in future studies. Future efforts by the global neuroscience community including the ENIGMA consortium [96–100], will be needed to aggregate the necessary large-scale individual multimodal neuroimaging, genetic, and behavioral data across multiple neurodevelopmental and psychiatric disorders to address these questions at the level of the individual.

In summary, we have identified cortical signatures of positive and negative schizotypy that are embedded along multiple scales of cortical organization and neuropsychiatric pathologies. More broadly, our work yields novel insights into how neurobiology and brain architecture may guide neuroanatomical vulnerability to general psychopathology and psychosis in the general population.

## Methods

### Study sample

This study included 2730 unmedicated healthy individuals with varying levels of positive and negative schizotypy (below) from the worldwide ENIGMA Schizotypy Working Group. Sample-size average of mean (range) age across samples for this meta-analysis was 30.1 (12–68) years and samples were on average 46.5% male (27–100%) (Table S1-S2). All participants were healthy individuals with no history of psychiatric or neurological disorders and were not taking any psychotropic medication. Each study sample was collected with participants’ informed consent and approved by local ethics review boards.

### Assessment of positive and negative schizotypy

Across all 31 cohorts, schizotypy was assessed with well-validated self-report instruments, including the Chapman scales [101, 102], the Community Assessment of Psychotic Experiences (CAPE) [103], the Schizotypal Personality Questionnaire (SPQ) [104], the Oxford-Liverpool Inventory of Feelings and Experiences (O-LIFE) [105], and the Rust Inventory of Schizotypal Cognitions (RISC) [106]. Positive schizotypy was assessed with the SPQ (or SPQ-B) cognitive/perceptual subscore, the CAPE positive dimension, the Chapman perceptual aberration score, the O-LIFE unusual experience score, and the RISC total score. Total negative schizotypy was assessed with the SPQ (SPQ-B) interpersonal subscore, the CAPE negative dimension, the Chapman physical and social anhedonia, and the O-LIFE introvertive anhedonia subscale (for details of schizotypy measures, see Table S4). In the case of two scores per site (London1a and Melbourne), the mean score of both scales was used.

### Image acquisition and processing

Following published ENIGMA procedures [31, 32, 107], all sites processed T1-weighted structural scans using FreeSurfer [52, 53] (http://surfer.nmr.mgh.harvard.edu) and extracted CT, SA for 68 Desikan-Killiany (DKT) atlas regions [51] (34 regions per hemisphere). In addition, subcortical volumes of 16 brain structures including left and right lateral ventricle, thalamus, caudate, putamen, pallidum, accumbens, hippocampus and amygdala, and intracranial volume (ICV) were extracted. Number of scanners, vendor, strength, sequence, acquisition parameters, and FreeSurfer versions are provided in Table S3. QC followed standard ENIGMA protocols at each site before analysis. For subcortical data, all regions of interest (ROIs) with a volume deviating from the mean by more than 1.5 times from the interquartile range were identified and only included after additional visual inspection. For cortical data, ENIGMA’s quality assurance protocol was performed (http://enigma.usc.edu/protocols/imaging-protocols) including visual inspection of the cortical segmentation and region-by-region removal of values from incorrect segmentations.

### Statistical meta-analyses

#### Model fit cortical measures

To examine the relationship between positive or negative schizotypy scores and CT (or SA) we fitted continuous models in each sample separately. We used partial correlation analysis (pcor.test, R version 3.6.0, R Foundation for Statistical Computing, Vienna, Austria) [108] to assess the association between the CT (or SA) of left and right DKT atlas regions with positive or negative schizotypy scores including age, sex as covariates. For multiscanner studies (n = 3), binary dummy covariates (n-1 scanners) were included within each local site model prior to meta-analysis to account for potential differences that may emerge across different scanners, consistent with the standard meta-analysis approach of previous ENIGMA studies [32, 107].

#### Model fit subcortical volumes

Similar to the cortical analyses, we applied continuous models to examine the relationship between schizotypy scores and subcortical volume for each ROI in each sample. To this end, partial correlation analysis was used to test correlations between the left and right subcortical volumes with positive and negative schizotypy scores including age, sex, and ICV as covariates. For multi-scanner studies (n = 3), binary dummy covariates were included to account for differences that may emerge across scanners, as described above.

#### Meta-analyses

Pearson’s r effect sizes from the partial correlations using schizotypy scores as a continuous predictor were meta-analyzed in separate random effects models to account for between study differences (rma function, metafor package for R 3.6.0) [109]. The false discovery rate (FDR) procedure (*p*_FDR_ < 0.05) was used to control for multiple comparisons across both hemispheres (n = 68) and for each schizotypy dimension separately [54]. Meta-analyses were adjusted for sample sizes across different sites and results were weighted for sample sizes. Possible confounding effects of schizotypy questionnaire type, FreeSurfer version, number of scanners, and scanner field strength were examined using moderator analyses (Tables S7-S12).

### Aggregation of the cortical profiles of positive and negative schizotypy within functional networks and cortical hierarchy levels

We stratified the regional effect sizes of cortical thickness associations with positive and negative schizotypy according to intrinsic functional brain networks [55] and levels of cortical hierarchy [56]. Following previous work, each DKT region was annotated to one of the seven functional network and four cortical hierarchy levels [34]. Mean cortical effect sizes were calculated separately for each network and for each level of the cortical hierarchy, based on the annotated individual DKT regions. We then calculated the mean t-values of the association between cortical thickness and positive or negative schizotypy for each functional network or cortical hierarchy. Significance of mean t-values in each functional network or cortical hierarchy was assessed with one sample t-test against 10000 null models using spin permutation testing to account for spatial autocorrelation [110, 111].

### Cortical pattern similarity of schizotypy dimensions with neurodevelopmental disorders

We examined how the spatial pattern of cortical differences in positive and negative schizotypy relate to the cortical alteration patterns observed in, CHR, schizophrenia, 22q11DS psychosis vs non-psychosis individuals, ADHD, and ASD. Similar to prior work [31, 33, 35, 36, 38, 77], we spatially correlated the cortical alteration patterns of positive and negative schizotypy (correlation coefficients) with the recently published ENIGMA meta-/mega-analyses of cortical abnormalities maps (Cohen’s *d*) of CHR [47], schizophrenia [32], 22q11DS psychosis [50], paediatric ADHD [49], ASD [48]. Specifically, we systematically compared the spatial pattern similarity of cortical effect size maps for positive and negative schizotypy with each of the other disorder-specific cortical effect size maps, using Pearson correlations. Statistical significance of all cortical pattern correlations was assessed using spin permutation tests (10000 repetitions) correcting for spatial autocorrelation [110, 112] implemented in the ENIGMA toolbox [111]. This spatial autocorrelation approach–and the adequate control of false positives–has been comprehensively validated for parcellated ROI data, including resolutions of 68 parcellations as in our study [112].

### Multiple-comparison correction for network/hierarchy and spatial similarity analyses

The aggregation analyses across functional networks and cortical hierarchy levels (i.e., the network/hierarchy enrichment analyses), as well as the cortical pattern-similarity analyses with neurodevelopmental disorders were performed to contextualize the spatial distribution and effect-size patterns of positive and negative schizotypy. Accordingly, our primary interpretive focus was on the spatial patterning and magnitude of effect sizes, with spin-permutation testing providing a spatially informed reference rather than serving as the primary basis for dichotomous statistical inference. In addition, we applied multiple-comparison correction to the spin permutation derived p-values. Specifically, within each schizotypy dimension we controlled the false discovery rate (FDR; Benjamini–Hochberg, q < 0.05) across the 11 enrichment tests (7 Yeo intrinsic networks and 4 Mesulam cortical hierarchy levels). For the cortical pattern-similarity analyses, we controlled FDR within each schizotypy dimension across the six disorder-specific cortical maps (n = 6). Thus, FDR correction was applied separately for positive and negative schizotypy in both the enrichment and spatial similarity analyses.

### Molecular, connectivity and neurotransmitter contributions to cortical morphology of schizotypy dimensions

We examined how cortical profiles in positive and negative schizotypy are informed by three different sets of molecular (or microarchitectural), normative structural connectivity, and specific neurotransmitter measures. All molecular, structural connectivity, and neurotransmitter maps provide regional values for each of the 68 DKT regions and were derived from two recent publications [36, 37]. Details on all molecular, connectivity, and neurotransmitter maps as well as dominance analysis can be found in the supplement as well as in the publications from Hansen and colleagues [36, 37]. Data can be obtained here: https://github.com/netneurolab/hansen_crossdisorder_vulnerability.git, https://github.com/netneurolab/hansen_receptors.git.

### Dominance analysis

Dominance analysis is a technique designed to assess the individual significance of each predictor within the multiple linear regression framework in terms of their contribution to the model’s overall goodness of fit, as indicated by the adjusted R² (see https://github.com/dominance-analysis/dominance-analysis and [113, 114] for detailed methodology). In this approach the same regression model is fitted on every combination of input variables (2p − 1 submodels for a model with p input variables). Total dominance is quantified as the mean increase in R² observed when a particular predictor is incorporated into a submodel, averaged over all possible submodel combinations, which amounts to 2^p - 1 variations for p predictors. The sum of the dominance of all input variables is equal to the total adjusted R^2^ of the full model, making total dominance an intuitive measure of contribution. To facilitate comparison across predictors, we report standardized dominance values, obtained by normalizing each predictor’s total dominance by the sum of total dominance across all predictors. These standardized dominance values range from 0 to 1 and sum to 1 within each model, matching the proportions shown in Fig. 4b.

### Hub vulnerability model

Following previous work [38, 40, 63], we tested the hub vulnerability hypothesis, *i.e*., that nodes with higher normative network centrality derived from an independent sample of healthy individuals of the HCP would display higher levels of cortical differences related to positive or negative schizotypy. To ensure spatial correspondence with derived cortical effect-size maps of positive and negative schizotypy all analyses were performed using the cortical parcellation of the DKT atlas. We note that structural network metrics, including hub identification, can vary across parcellations [95], which we address in the study limitations. Using the analyses pipeline of the ENIGMA toolbox [111], we assessed spatial correlations between the cortical morphology profiles of positive and negative schizotypy and normative weighted degree centrality maps (Fig. S2, S3). Weighted degree centrality was defined by the sum of all weighted cortico-cortical connections for every region and used to identify structural and functional hub regions. Regions with a higher weighted degree centrality are denoted as hubs, compared to regions with relatively lower weighted degree centrality. To mitigate potential bias from selecting an arbitrary threshold and inadvertently excluding valuable information, the analyses were conducted on unthresholded connectivity matrices. We corrected for spatial autocorrelation using the spin permutation procedure (10000 repetitions) implemented in the ENIGMA toolbox [111] (see above).

### Mapping disease epicenters

Similar to the hub vulnerability model, we followed previous work [36, 38, 40, 43, 44, 63] and aimed to identify epicenters of cortical morphology patterns related to positive or negative schizotypy. Epicenters are defined as regions whose normative connectivity profile most closely resembles disease-related morphological alteration patterns [36, 38, 40, 44, 45, 63]. In other words, disease epicenters are connected to those brain regions with the strongest disease-related morphological alterations. We spatially correlated every region’s healthy functional and structural cortical connectivity profile (Fig. S1, S2) to the whole-brain patterns of cortical differences in positive and negative schizotypy (Fig. 2). We repeated this approach systematically for each parcellated region and for functional and structural cortical connectivity separately. Regions were ranked in descending order based on the strength of their correlation coefficients, with the highest-ranked regions being considered the most significant disease epicenters. A region can be considered an epicenter regardless of its cortical difference magnitude (correlation coefficient of the partial correlations with either positive or negative schizotypy), as long as it is strongly connected to other regions with high cortical differences (higher *r*-values) and weakly connected to regions with low cortical alteration (*r*-values). Statistical significance of spatial similarity between an individual brain region’s functional and structural connectivity profile and schizophrenia-related cortical alterations was determined using the spin permutation procedure (1000 repetitions) implemented in the ENIGMA toolbox [111] (see above). To verify the robustness of the epicenter results, we additionally evaluated statistical significance using a complementary network-rewiring null model that preserves degree and distance relationships of the connectome [36, 44, 115, 116]. Implementation details and results are provided in the supplement and Fig. S4.

## Supplementary information

Supplement

## Data Availability

All meta-analytic data reported in the manuscript are included in the article and its supplementary information files. Summary statistics of the case-control meta-analyses from the ENIGMA Working Groups are available from the ENIGMA Toolbox (https://enigma-toolbox.readthedocs.io/en/latest/). Preprocessed normative cortico-cortical, subcortico-cortical, and subcortico-subcortical functional and structural connectivity data are available from the ENIGMA Toolbox (https://enigma-toolbox.readthedocs.io/en/latest/). Molecular, connectivity, and receptor datasets are available from https://github.com/netneurolab/hansen_crossdisorder_vulnerability.git and https://github.com/netneurolab/hansen_receptors.git. Additional information can be made available upon reasonable request to the authors.

## Code availability

All software and analysis code used in this study are openly available. Image preprocessing pipelines are available through the ENIGMA consortium (https://github.com/ENIGMA-git). Statistical analyses were performed in R (version 3.6.0), including partial correlation analyses using pcor.test and random-effects meta-analyses using the metafor package (rma function). Spatial correlation, hub, and epicenter analyses were conducted using the ENIGMA Toolbox (https://enigma-toolbox.readthedocs.io/en/latest/). Dominance analysis was performed with the domir package (version 1.2.0; 10.32614/CRAN.package.domir).
