# Supplementary material for: Multiscale characterization of cortical signatures in positive and negative schizotypy: a worldwide ENIGMA study
Source: Mol Psychiatry. 2026 Apr 18;31(9):4846–60. doi: 10.1038/s41380-026-03547-x (PMC13441894; doi:10.1038/s41380-026-03547-x)
Supplement: Supplementary file 1 [file 41380_2026_3547_MOESM1_ESM.docx]

**Multiscale characterization of cortical signatures in positive and negative schizotypy: A worldwide ENIGMA study**

Supplement

**Table of Contents**

[Table S1. Demographics sites included in positive schizotypy analysis	2](#_Toc214893507)

[Table S2. Demographics, sites included in negative schizotypy analysis	3](#_Toc214893508)

[Table S3. Scanner Details	4](#_Toc214893509)

[Table S4. Questionnaires/Rating Scales used across sites	8](#_Toc214893510)

[Table S5. Association between CT and positive schizotypy corrected for age, and sex	9](#_Toc214893511)

[Table S6. Association between CT and negative schizotypy corrected for age, and sex	11](#_Toc214893512)

[Table S8. Freesurfer version moderator of association with CT and positive schizotypy	15](#_Toc214893513)

[Table S9. Field strength moderator of association with CT and positive schizotypy	17](#_Toc214893514)

[Table S10. Negative schizotypy questionnaire moderator CT and negative schizotypy association	19](#_Toc214893515)

[Table S11. Freesurfer version moderator of association with CT and negative schizotypy	21](#_Toc214893516)

[Table S12. Field strength moderator of association with CT and negative schizotypy	23](#_Toc214893517)

[Table S13. Association between SA and positive schizotypy corrected for age, and sex	25](#_Toc214893518)

[Table S14. Association between SA and negative schizotypy corrected for age, and sex	27](#_Toc214893519)

[Table S15. Association between SV and positive schizotypy corrected for age, and sex	29](#_Toc214893520)

[Table S16. Association between SV and negative schizotypy corrected for age, and sex	30](#_Toc214893521)

[Fig S1. Associations between CT profiles of positive and negative schizotypy with those from CHR stages and schizophrenia	31](#_Toc214893522)

[Molecular, connectivity and neurotransmitter contributions to cortical morphology of schizotypy dimensions	32](#_Toc214893523)

[Fig S2. Normative functional degree centrality derived from the HCP sample (n=207)	34](#_Toc214893524)

[Fig S3. Normative structural degree centrality derived from the HCP sample (n=207)	35](#_Toc214893525)

[Table S17. Functional epicenters of associations between CT and positive schizotypy	36](#_Toc214893526)

[Table S18. Structural epicenters of associations between CT and positive schizotypy	38](#_Toc214893527)

[Table S19. Functional epicenters of associations between CT and negative schizotypy	40](#_Toc214893528)

[Table S20. Structural epicenters of associations between CT and negative schizotypy	42](#_Toc214893529)

[Comparison epicenter analysis with spin-permutation null model and network-rewiring null model	44](#_Toc214893530)

[Fig S4. Epicenters derived from spin-permutation and network-rewiring null model	45](#_Toc214893531)

[Funding and Acknowledgements	46](#_Toc214893532)

[References	47](#_Toc214893533)

# Table S1. Demographics sites included in positive schizotypy analysis

| Site | N | Scale 1 | Scale 2 | %Male | Mean Age | Age SD (years) | Score 1 Mean | Score 1 SD | Score 2 Mean | Score 2 SD |
| --- | --- | --- | --- | --- | --- | --- | --- | --- | --- | --- |
| ASRB | 191 | SPQ-CP |  | 47.64 | 39.69 | 13.77 | 3 | 3.9 |  |  |
| AUK | 49 | OLIFE-UE |  | 34.69 | 23.33 | 4.5 | 12.51 | 6.84 |  |  |
| BEIJ-ECNU | 70 | CHAPMAN-PA |  | 45.71 | 19.89 | 1.16 | 14.28 | 6.42 |  |  |
| BEIJ-GZ | 76 | CHAPMAN-PA |  | 48.68 | 19.28 | 0.9 | 15.62 | 9.61 |  |  |
| BONN1 | 126 | SPQ-CP |  | 51.97 | 27 | 7.84 | 3 | 2.5 |  |  |
| BONN2 | 51 | RISC |  | 64.71 | 27.2 | 5.36 | 22.37 | 7.71 |  |  |
| BONN3 | 31 | OLIFE-UE |  | 43.75 | 24 | 4.52 | 5 | 3.73 |  |  |
| CAM | 89 | SPQ-CP |  | 46.07 | 24.07 | 5.3 | 5.54 | 4.93 |  |  |
| NYC | 240 | CAPE-POS | SPQ-CP | 51.33 | 35.99 | 12.94 | 22.67 | 3.14 | 1.89 | 3.05 |
| FIDMAG | 64 | RISC |  | 42.19 | 37.89 | 10.29 | 22.5 | 9.91 |  |  |
| GENEVA | 114 | SPQ-CP |  | 54.63 | 16.43 | 2.63 | 8.49 | 6.87 |  |  |
| GRON1 | 58 | PANSS-POS |  | 73.13 | 34.23 | 10.99 | 14.35 | 4.29 |  |  |
| GRON2 | 43 | SPQ-CP |  | 62.79 | 22.67 | 2.26 | 7.59 | 5.91 |  |  |
| IGP | 63 | SPQ-CP |  | 55.56 | 36.43 | 11.05 | 4.05 | 5.03 |  |  |
| LOND1a | 46 | SPQ-CP | OLIFE-UE | 52.17 | 27.46 | 6.84 | 5.3 | 6.67 | 6.02 | 6.4 |
| LOND1b | 40 | CAPE-POS |  | 52.5 | 20.8 | 3.76 | 1.44 | 0.34 |  |  |
| LOND2 | 44 | OLIFE-UE |  | 27.27 | 29.41 | 9.2 | 3.27 | 3.44 |  |  |
| LOND3 | 40 | SPQ-CP |  | 60 | 30.97 | 10.87 | 2.45 | 2.07 |  |  |
| LOND4 | 26 | CAPE-POS |  | 65.38 | 38.62 | 7.34 | 28.15 | 6.27 |  |  |
| FOR-2107-MR | 446 | SPQ-CP |  | 37.44 | 34.67 | 12.85 | 0.83 | 0.89 |  |  |
| MELB | 376 | CAPE-POS | OLIFE-UE | 43.35 | 23.39 | 5.15 | 25.85 | 4.68 | 2.98 | 2.65 |
| MTL | 40 | CHAPMAN-PA |  | 28 | 19.97 | 1.25 | 13.6 | 9.28 |  |  |
| MOSC | 107 | SPQ-CP |  | 48.6 | 26.46 | 6.61 | 6.18 | 4.67 |  |  |
| MNC | 173 | SPQ-CP |  | 46.82 | 43.31 | 11.85 | 1.46 | 1.45 |  |  |
| FOR-2017-MS | 228 | SPQ-CP |  | 35.96 | 28.42 | 10.41 | 0.7 | 0.83 |  |  |
| PARIS | 52 | SPQ-CP |  | 42.31 | 35.85 | 11.24 | 1.32 | 2.23 |  |  |
| ZUR1 | 27 | SPQ-CP |  | 62.96 | 29.15 | 10.35 | 17.56 | 5.01 |  |  |
| ZUR2 | 62 | SPQ-CP |  | 100 | 27.74 | 4.74 | 8.76 | 6.45 |  |  |

# Table S2. Demographics, sites included in negative schizotypy analysis

| Site | N | Scale 1 | Scale 2 | % male | Mean age | Age SD | Score 1 Mean | Score 1 SD | Score 2 Mean | Score 2 SD |
| --- | --- | --- | --- | --- | --- | --- | --- | --- | --- | --- |
| AMS | 49 | CAPE |  | 53.06 | 21.36 | 3.08 | 45.51 | 9.90 |  |  |
| ASRB | 191 | SPQ |  | 52.36 | 39.69 | 13.77 | 5.23 | 5.46 |  |  |
| AUK | 49 | OLIFE |  | 34.69 | 23.33 | 4.50 | 7.73 | 5.66 |  |  |
| BEIJ | 146 | SPQ | CAP | 47.26 | 19.57 | 1.08 | 18.56 | 10.08 | 9.97 | 7.54 |
| BONN1 | 93 | SPQ |  | 50.54 | 28.08 | 8.02 | 2.81 | 2.93 |  |  |
| CAM | 88 | SPQ |  | 45.45 | 24.33 | 4.72 | 5.45 | 4.41 |  |  |
| FOR-2017-MS | 228 | SPQB |  | 35.96 | 28.42 | 10.41 | 1.49 | 1.65 |  |  |
| FOR-2107-MR | 446 | SPQB |  | 37.44 | 34.67 | 12.85 | 1.78 | 1.74 |  |  |
| GENEVA | 114 | SPQ |  | 52.63 | 15.94 | 2.64 | 6.04 | 4.31 |  |  |
| GRON1 | 35 | CAPE-Adj |  | 51.43 | 36.11 | 9.31 | 6.41 | 1.29 |  |  |
| GRON2 | 42 | SPQB |  | 61.9 | 22.67 | 2.26 | 5.55 | 4.43 |  |  |
| IGP | 63 | SPQB |  | 55.56 | 36.43 | 11.05 | 6.44 | 6.00 |  |  |
| LOND1a | 46 | SPQB | OLIFE | 52.17 | 27.48 | 6.83 | 6.18 | 5.41 | 13.09 | 3.02 |
| LOND3 | 40 | SPQB |  | 60 | 30.97 | 10.87 | 3.45 | 2.32 |  |  |
| LOND4 | 26 | CAPE |  | 65.38 | 38.62 | 7.34 | 23.88 | 5.35 |  |  |
| MELB | 397 | CAPE |  | 43.32 | 23.42 | 5.17 | 23.81 | 5.71 |  |  |
| MNC | 173 | SPQB |  | 46.82 | 43.31 | 11.85 | 3.34 | 3.08 |  |  |
| MOSC | 58 | SPQ |  | 51.72 | 25.66 | 5.13 | 6.93 | 6.35 |  |  |
| MTL | 40 | CHAP |  | 32.5 | 19.83 | 1.14 | 15.70 | 11.28 |  |  |
| NYC | 225 | CAPE-Adj |  | 51.11 | 35.92 | 12.85 | 1.25 | 0.26 |  |  |
| UTR | 91 | SPQ |  | 37.36 | 40.92 | 14.26 | 5.82 | 6.50 |  |  |
| ZUR1 | 27 | SPQ |  | 62.96 | 29.15 | 10.35 | 18.15 | 5.40 |  |  |
| ZUR2 | 62 | SPQ | CHAP | 100 | 27.74 | 4.74 | 7.03 | 4.95 | 10.90 | 5.78 |

# Table S3. Scanner Details

| **Sample** | **Number of Scanners** | **Scanner Vendor & Type** | **Imaging Protocols** | **Slice Orientation** | **FreeSurfer Version** | **Operating System** | **Number of subjects removed after QC** |
| --- | --- | --- | --- | --- | --- | --- | --- |
| AMS | 1 | 3T Phillips Achieva | 3D T1-weighted images (TR = 8.2, TE = 3.8, FA = 8°, FOV 240 × 188 mm, voxel size 1 × 1 × 1, 220 slices) | sequential ascending | 6.0.1 | virtual linux environment (sigularity on HPC) | 0 |
| ASRB | 5 | 1.5T Siemens Avanto | 3D MPRAGE; TR 1980ms, TE 4.3ms, field-of-view 250 x 250 mm2, data acquisition matrix 256 x 256, 176 contiguous 1mm slices; voxel size 0.98 x 0.98 x 1.0 mm3, flip angle 15° | sagittal | 5.1 | Mac OS X 10.9.5 | 0 |
| AUCK | 1 | 3T Siemens | 3D MPRAGE; FOV = 256 mm2; matrix size = 256x 256 mm; number of slices = 176; slice thickness = 1 mm; voxel-size = 1 x1 x1 mm3; TE/TR/flip angle = 2.07 ms/1900 ms/9 deg; GRAPPA acceleration factor = 2; TA = 4.26 min. | interleaved | 5.3.0 | linux | 0 |
| BEIJ | 2 | 3T Siemens | site 1: MPRAGE; TR 2530ms, TE 2.34ms, FOV 256mm, matrix 256x256, 192 slices, slice thickness 1mm, flip angle 7; site 2: MPRAGE; TR 2530ms, TE 2.34ms, FOV 256mm, matrix 256x256, 172 slices, slice thickness 1mm, flip angle 7; | sagittal | 6.0.0 | Linux | 0 |
| BONN_3 | 1 | 3T Siemens Trio | TR = 1570 ms; TE = 3.42 ms; inversion time (TI) = 800 ms; flip angle = 15°; FoV = 256 mm; matrix size = 256 x 256; 160 slices; slice thickness = 1 mm; voxel size = 1 x 1 x 1 | sequential | 6.0.0 |  | 0 |
| BONN_2 | 2 | 1.5 T GE Signa Advantage, 3T Siemens Magnetom Verio | GE Signa Advantage: 3D-SPGR voxel resolution: 1 x 1 x 1.5 mm; repetition time (TR): 18 ms; inversion time (TI): 450 ms; echo time (TE): 5.1 ms; bandwidth: 15.63 kHz |  | 6.0.0 |  | 0 |
| BONN1 | 1 | 3T Siemens Magnetom Verio | 3D MPRAGE sequence, repetition time of TR = 2400 ms, echo time TE = 3.06 ms, flip angle = 9 degrees with 160 slices, slice thickness = 1.0 mm, voxel size = 1.0 × 1.0 × 1.0 mm, field of view FOV = 256 mm |  | 6.0.0 |  | 0 |
| CAM | 1 | 3T Siemens Trio | MPRAGE; TR/TE 2.98/2300 ms, 1 × 1 voxels, slice thickness 1 mm, flip angle 9°, FOV 24 × 25.6 mm, 176 slices |  | 6.0.0 | Unix | 0 |
| FIDMAG | 1 | 1.5T GE Signa Excite | 3D TR=12.356 ms, TE=5.192 ms, inversion time=450 ms, acquisition matrix=256 x 224, voxel size=1*1*1 mm^3, flip angle=20°, 180 slices | axial | 7.1.1 | Linux | 0 |
| FOR2107-MR | 1 | 3T Siemens Magnetom TiroTim syngo | 3D T1-weighted magnetization prepared rapid acquisition gradient echo (MPRAGE); TR=1900ms, TE=2.26ms, TI=900ms, FA=9°, voxel size=1.0x1.0x1.0mm³, Acquisition Direction Sagittal, 176 slices, slice gap 0.5mm. | Sagittal | 5.3 | Red Hat Enterprise Linux Server release 5.11 (Tikanga) | 0 |
| FOR2107-MS | 1 | 3T Siemens PRISMA | 3D T1-weighted magnetization prepared rapid acquisition gradient echo (MPRAGE); TR=2130ms, TE=2.28ms, TI=900ms, FA=8°, voxel size=1.0x1.0x1.0mm³, Acquisition Direction Sagittal, 192 slices, no slice gap. | Sagittal | 5.3 | Red Hat Enterprise Linux Server release 5.11 (Tikanga) | 0 |
| GENEVA | 2 | 3T Siemens Trio | T1-Weighted images (192 slices, TR=2500ms, TE=3ms, flip angle= 8 degree, slice thickness=1.1mm, FOV=22cm, Acq matrix 256x256) | A >> P or descending | 6.0.0 | Mac OS SIERRA v 10.12.6 | 0 |
| GRON1 | 1 | 3T Phillips | a T1-weighted image was obtained (TR/TE = 9/3.5 ms) using fast-field echo and turbo-field echo: 170 axial slices; FOV (rl, ap, fh) = 232 × 170 × 256 mm; flip angle = 8°, voxel size = 1 × 1 × 1 mm, slice thickness = 1 mm. | descending | 6.0.1 | virtual linux environment (sigularity on HPC) | 0 |
| GRON2 | 1 | 3T Phillips | anatomic images were obtained using a sagittal 3-dimensional T1-weighted sequence (176 slices, repetition time 9 ms, echo time 3.5 ms, field of view 256 mm, voxel size 1 × 1 × 1 mm, slice thickness 1.0 mm) using an 8 channel head coil. | descending | 6.0.1 | virtual linux environment (sigularity on HPC) | 0 |
| IGP | 1 | 3T Phillips Achieva TX | 3D MPRAGE; TR 8.9ms, TE 4.1ms, field of view 240mm, matrix 268 x 268, 200 slices, slice thickness 0.9mm, no gap | sagittal | 5.3 | Mac OS X 10.9.5 | 0 |
| JENA1 | 1 | 3T Siemens Tim Trio | 3D T1-weighted MPRAGE, TR: 2300ms, TE: 3.03 ms, flip angle: 9°, 192 slices, field of view 256mm, voxel resolution 1x1x1mm, acquisition time: 5:21min | sagittal | 6.0.0 | Linux | 0 |
| LOND1a | 1 | 3T GE Discovery MR750 | 3D T1-weighted inversion recovery prepared gradient echo sequence (voxel size: 1.05 × 1.05 × 1.2 mm, field of view: 270 mm, 196 slices, TR: 7.3 ms, TE: 3.0 ms, inversion time: 400 ms, flip angle = 11°), based on the well-validated ADNI 2/ADNI GO protocols (see http://adni.loni.usc.edu/methods/documents/mri-protocols/). | sequential (top down) | 6.0.0 | Unix | 0 |
| LOND1b | 1 | 3T Phillips Intera | T1-weighted 3D fast-field echo (FFE) sequence [repetition time (TR)=25 ms, echo time (TE)=4.6 ms, field of view (FOV)=260 mm, matrix=256x256, 160 contiguous axial slices of 1-mm thickness, voxel size= 1x1x1 mm]. | interleaved | 6.0.0 | Unix | 0 |
| LOND2 | 2 | 1.5 T GE, 3T GE | 1.5 T GE: 3D, TR=11.1 ms, TE=4.9 ms, inversion time=300 ms, acquisition matrix=256 x 160, 150 locations, slice thickness=1.1 mm, in-plane resolution=1.094 mm, flip angle=18°; 3 T GE: TR: 7.3 ms, TE: 3.0 ms, inversion time: 400 ms, voxel size: 1.05 x1.05 x1.2mm, field of view: 270mm, 196 slices, flip angle = 11° |  | 6.0.0 | Unix | 0 |
| LOND3 | 1 | 3T Siemens | MPRAGE; TR 2000ms; 1mm x 1mm 1mm. 256 x 256 x 176 slices, flip angle 11, slice thickness 1mm, TE = 2.07ms | interleaved | 6.0.0 | Mac | 0 |
| LOND4 | 1 | 3T GE | T1-weighted image (196 slices; isotropic voxels of 1.2 mm; TR 7.312 ms; TE 3.016 ms;flip angle: 11°; FOV 270 mm) | sequential (top down) | 6.0.0 | Unix | 0 |
| LOND5 | 1 | 3T Siemens | MPRAGE; TR 1900ms; 1mm x 1mm 1mm voxel size; in plane resolution of 256 x 256 x 176 slices, flip angle 11, slice thickness 1mm, TE = 2.07ms | interleaved | 6.0.0 | Linux Ubuntu Bionic | 0 |
| MELB | 1 | 3T Siemens | 3D magnetic-prepared rapid gradient echo sequence. A total of 192 slices were acquired for each participant’s T1-weighted images using an ascending acquisition with the following parameters: TR of 2300 ms, TE of 2.07 ms, flip angle of 9°, FOV of 256mm, and voxel size of 1mm3. | sagittal | 5.3.0 | Linux | 34 |
| MNC | 1 | 3T scanner Gyroscan Intera, Philips Medical Systems | 3D fast gradient echo sequence (turbo field echo), TR = 7.4 msec, TE = 3.4 msec, Flip Angle = 9°, two signal averages, inversion prepulse every 814.5 msec, acquired over a FOV of 256 (feet-head [FH]) × 204 (anterior-posterior [AP]) × 160 (right-left [RL]) mm, phase encoding in AP and RL direction, reconstructed to cubic voxels of .5 × .5 × .5 mm | Sagittal | 5.3 | Red Hat Enterprise Linux Server release 5.11 (Tikanga) | 0 |
| MOSC | 1 | 3 T Phillips Ingenia | 3D T1-weighted TFE; TR=7.9 ms, TE=3.5 ms; flip angle 8; number of slices=170; voxel size=0.98 x 0.98 x 1.0, no gap | axial | 5.3 | LinuxCentOS 6.10 | 0 |
| MTL | 1 | 3T Siemens Trio | 3D T1-weighted images were acquired with a gradient echo T1-weighted sequence, voxel size 1x1x1 mm3. TR=1900ms; TE=4.9ms; FA=25; matrix 176x256x256 | sagittal | 6.0.0 | Unix | 0 |
| NYC (Hillside) | 1 | 3T GE | 3D-SPGR images using a 1mm thick slice acquisition with image parameters: TR = 7.5 ms, TE = 3 ms, matrix = 256x256, FOV = 240 mm, 216 contiguous images. | interleaved | 6.0.0 | Linux | 0 |
| PARIS | 1 | 3T Siemens Trio | 3D T1 TE, 2.98 ms; TR, 2300 ms; 160 sections; voxel size, 1.0 × 1.0 × 1.1 mm |  |  |  |  |
| UTR | 1 | 3T Phillips Achieva | T1-weighted images were acquired with a 3D T1 turbo field-echo sequence. The acquisition protocol parameters were: 160 slices; repetition time = 9.96 ms; echo time = 4.59 ms; flip angle 8°; 1 mm slice thickness with no inter-slice gap; matrix = 256 × 256 and field of view 224 mm, achieving a voxel size of 0.875 × 0.875 × 1 mm. Scan time was 8 min and 50 s | saggital | 5.1.0 | Unix | 0 |
| ZUR1 | 1 | 3T Phillips | 3D T1-weighted images were acquired with an ultrafast gradient echo T1-weighted sequence (TR=8.4ms, TE=3.8ms, flip angle=8°) in 160 sagittal plan slices (1mm slice thickness, no slice gap) of 240×240mm2 resulting in 1x1x1mm3 voxels. | sagittal | 6.0.0 | Unix | 0 |
| ZUR2 | 1 | 3T Phillips Achieva | 3D T1-weighted images; 160 slices; TR, 8.2 ms; TE, 3.8 ms; flip angle, 8°; spatial resolution, 1 × 1 × 1 mm3; FOV = 160 × 240 mm2 | sagittal | 6.0.0 | OS X | 0 |

# Table S4. Questionnaires/Rating Scales used across sites

| Questionnaires | Total number of items | Factors/Subscales |
| --- | --- | --- |
| CAPE | 42 | Depression, Negative, Positive Symptoms |
| CHAP | 196 | Perceptual Aberration; Magical Ideation; Physical Anhedonia; Social Anhedonia; Impulsive Nonconformity |
| OLIFE | 104 | Unusual Experiences; Cognitive Disorganization;  Introvertive Anhedonia; Impulsive Nonconformity |
| PANSS | 30 | Positive; Negative; General symptoms |
| RISC | 26 | Positive and Cognitive content of schizotypy |
| SPQ | 74 | Cognitive/Perceptual; Interpersonal; Disorganized |
| SPQ-B | 22 | Cognitive/Perceptual; Interpersonal; Disorganized |

# Table S5. Association between CT and positive schizotypy corrected for age, and sex

| DKT Regions | Partial R | Std. Err. | 95% CI, lower bound | 95% CI, upper bound | p-value | FDR-pvalue | Sample size per region |
| --- | --- | --- | --- | --- | --- | --- | --- |
| L_bankssts_thickavg | -0.031 | 0.032 | -0.094 | 0.033 | 0.344 | 0.517 | 2539 |
| L_caudalanteriorcingulate_thickavg | 0.024 | 0.020 | -0.014 | 0.062 | 0.216 | 0.401 | 2704 |
| L_caudalmiddlefrontal_thickavg | -0.038 | 0.025 | -0.087 | 0.011 | 0.126 | 0.316 | 2711 |
| L_cuneus_thickavg | -0.045 | 0.026 | -0.096 | 0.007 | 0.092 | 0.271 | 2587 |
| L_entorhinal_thickavg | -0.021 | 0.028 | -0.076 | 0.035 | 0.467 | 0.615 | 2558 |
| L_fusiform_thickavg | -0.028 | 0.023 | -0.073 | 0.017 | 0.227 | 0.405 | 2676 |
| L_inferiorparietal_thickavg | -0.046 | 0.019 | -0.084 | -0.008 | 0.018 | 0.197 | 2634 |
| L_inferiortemporal_thickavg | 0.000 | 0.021 | -0.041 | 0.040 | 0.989 | 0.999 | 2651 |
| L_isthmuscingulate_thickavg | -0.026 | 0.019 | -0.063 | 0.012 | 0.175 | 0.380 | 2721 |
| L_lateraloccipital_thickavg | -0.028 | 0.019 | -0.065 | 0.010 | 0.151 | 0.351 | 2688 |
| L_lateralorbitofrontal_thickavg | -0.053 | 0.030 | -0.112 | 0.007 | 0.082 | 0.259 | 2715 |
| L_lingual_thickavg | -0.049 | 0.022 | -0.093 | -0.005 | 0.029 | 0.224 | 2672 |
| L_medialorbitofrontal_thickavg | 0.043 | 0.019 | 0.005 | 0.080 | 0.025 | 0.212 | 2689 |
| L_middletemporal_thickavg | 0.000 | 0.020 | -0.038 | 0.039 | 0.984 | 0.999 | 2546 |
| L_parahippocampal_thickavg | -0.004 | 0.037 | -0.078 | 0.069 | 0.909 | 0.992 | 2706 |
| L_paracentral_thickavg | -0.051 | 0.025 | -0.100 | -0.001 | 0.044 | 0.238 | 2714 |
| L_parsopercularis_thickavg | -0.071 | 0.019 | -0.109 | -0.034 | 0.000 | 0.018 | 2692 |
| L_parsorbitalis_thickavg | -0.063 | 0.019 | -0.101 | -0.026 | 0.001 | 0.043 | 2699 |
| L_parstriangularis_thickavg | -0.032 | 0.029 | -0.090 | 0.025 | 0.271 | 0.452 | 2685 |
| L_pericalcarine_thickavg | -0.042 | 0.026 | -0.093 | 0.009 | 0.106 | 0.287 | 2643 |
| L_postcentral_thickavg | -0.074 | 0.032 | -0.137 | -0.012 | 0.020 | 0.197 | 2665 |
| L_posteriorcingulate_thickavg | -0.005 | 0.025 | -0.055 | 0.045 | 0.838 | 0.952 | 2720 |
| L_precentral_thickavg | -0.060 | 0.025 | -0.109 | -0.010 | 0.018 | 0.197 | 2686 |
| L_precuneus_thickavg | -0.024 | 0.026 | -0.074 | 0.027 | 0.356 | 0.518 | 2710 |
| L_rostralanteriorcingulate_thickavg | 0.024 | 0.019 | -0.013 | 0.062 | 0.201 | 0.401 | 2695 |
| L_rostralmiddlefrontal_thickavg | -0.035 | 0.019 | -0.072 | 0.003 | 0.071 | 0.238 | 2700 |
| L_superiorfrontal_thickavg | -0.038 | 0.019 | -0.075 | 0.000 | 0.050 | 0.238 | 2699 |
| L_superiorparietal_thickavg | -0.051 | 0.027 | -0.104 | 0.001 | 0.056 | 0.238 | 2665 |
| L_superiortemporal_thickavg | -0.023 | 0.025 | -0.072 | 0.025 | 0.347 | 0.517 | 2529 |
| L_supramarginal_thickavg | -0.043 | 0.022 | -0.087 | 0.000 | 0.049 | 0.238 | 2584 |
| L_frontalpole_thickavg | 0.001 | 0.019 | -0.036 | 0.038 | 0.955 | 0.995 | 2726 |
| L_temporalpole_thickavg | 0.022 | 0.028 | -0.032 | 0.077 | 0.417 | 0.571 | 2679 |
| L_transversetemporal_thickavg | 0.009 | 0.031 | -0.052 | 0.070 | 0.766 | 0.896 | 2726 |
| L_insula_thickavg | -0.027 | 0.022 | -0.071 | 0.016 | 0.220 | 0.401 | 2673 |
| R_bankssts_thickavg | -0.002 | 0.019 | -0.040 | 0.036 | 0.921 | 0.992 | 2658 |
| R_caudalanteriorcingulate_thickavg | 0.009 | 0.019 | -0.029 | 0.046 | 0.646 | 0.798 | 2716 |
| R_caudalmiddlefrontal_thickavg | -0.040 | 0.024 | -0.086 | 0.006 | 0.089 | 0.271 | 2700 |
| R_cuneus_thickavg | -0.030 | 0.027 | -0.082 | 0.022 | 0.265 | 0.449 | 2613 |
| R_entorhinal_thickavg | 0.012 | 0.028 | -0.043 | 0.066 | 0.671 | 0.801 | 2472 |
| R_fusiform_thickavg | 0.000 | 0.019 | -0.038 | 0.038 | 0.999 | 0.999 | 2682 |
| R_inferiorparietal_thickavg | -0.033 | 0.027 | -0.086 | 0.020 | 0.217 | 0.401 | 2628 |
| R_inferiortemporal_thickavg | -0.028 | 0.019 | -0.066 | 0.010 | 0.148 | 0.351 | 2659 |
| R_isthmuscingulate_thickavg | -0.005 | 0.019 | -0.042 | 0.033 | 0.813 | 0.935 | 2711 |
| R_lateraloccipital_thickavg | -0.064 | 0.028 | -0.120 | -0.009 | 0.024 | 0.212 | 2677 |
| R_lateralorbitofrontal_thickavg | -0.023 | 0.038 | -0.096 | 0.051 | 0.542 | 0.678 | 2694 |
| R_lingual_thickavg | -0.041 | 0.035 | -0.109 | 0.027 | 0.242 | 0.417 | 2671 |
| R_medialorbitofrontal_thickavg | -0.006 | 0.020 | -0.046 | 0.034 | 0.770 | 0.896 | 2667 |
| R_middletemporal_thickavg | 0.020 | 0.030 | -0.039 | 0.078 | 0.506 | 0.649 | 2637 |
| R_parahippocampal_thickavg | -0.045 | 0.028 | -0.101 | 0.010 | 0.107 | 0.287 | 2708 |
| R_paracentral_thickavg | -0.037 | 0.020 | -0.077 | 0.002 | 0.060 | 0.238 | 2713 |
| R_parsopercularis_thickavg | 0.001 | 0.019 | -0.037 | 0.038 | 0.973 | 0.999 | 2666 |
| R_parsorbitalis_thickavg | -0.045 | 0.028 | -0.101 | 0.010 | 0.110 | 0.287 | 2695 |
| R_parstriangularis_thickavg | -0.043 | 0.025 | -0.093 | 0.006 | 0.083 | 0.259 | 2661 |
| R_pericalcarine_thickavg | -0.017 | 0.040 | -0.096 | 0.062 | 0.669 | 0.801 | 2625 |
| R_postcentral_thickavg | -0.068 | 0.024 | -0.114 | -0.021 | 0.004 | 0.086 | 2665 |
| R_posteriorcingulate_thickavg | 0.017 | 0.019 | -0.021 | 0.054 | 0.387 | 0.545 | 2721 |
| R_precentral_thickavg | -0.066 | 0.032 | -0.128 | -0.003 | 0.041 | 0.238 | 2668 |
| R_precuneus_thickavg | -0.027 | 0.022 | -0.071 | 0.016 | 0.221 | 0.401 | 2709 |
| R_rostralanteriorcingulate_thickavg | 0.026 | 0.032 | -0.036 | 0.088 | 0.408 | 0.567 | 2662 |
| R_rostralmiddlefrontal_thickavg | -0.027 | 0.019 | -0.064 | 0.011 | 0.167 | 0.370 | 2678 |
| R_superiorfrontal_thickavg | -0.031 | 0.019 | -0.068 | 0.006 | 0.104 | 0.287 | 2699 |
| R_superiorparietal_thickavg | -0.054 | 0.029 | -0.111 | 0.004 | 0.066 | 0.238 | 2676 |
| R_superiortemporal_thickavg | -0.011 | 0.026 | -0.063 | 0.041 | 0.673 | 0.801 | 2611 |
| R_supramarginal_thickavg | -0.024 | 0.027 | -0.076 | 0.028 | 0.374 | 0.534 | 2601 |
| R_frontalpole_thickavg | 0.025 | 0.019 | -0.012 | 0.063 | 0.190 | 0.401 | 2716 |
| R_temporalpole_thickavg | -0.002 | 0.020 | -0.041 | 0.038 | 0.933 | 0.992 | 2612 |
| R_transversetemporal_thickavg | -0.022 | 0.019 | -0.060 | 0.015 | 0.241 | 0.417 | 2729 |
| R_insula_thickavg | 0.003 | 0.036 | -0.067 | 0.073 | 0.928 | 0.992 | 2640 |

# Table S6. Association between CT and negative schizotypy corrected for age, and sex

| DKT Regions | Partial R | Std. Err. | 95% CI, lower bound | 95% CI, upper bound | p-value | FDR-pvalue | Sample size per region |
| --- | --- | --- | --- | --- | --- | --- | --- |
| L_bankssts_thickavg | 0.007 | 0.020 | -0.032 | 0.046 | 0.709 | 0.973 | 2512 |
| L_caudalanteriorcingulate_thickavg | 0.013 | 0.019 | -0.025 | 0.050 | 0.514 | 0.971 | 2668 |
| L_caudalmiddlefrontal_thickavg | 0.031 | 0.029 | -0.025 | 0.087 | 0.282 | 0.784 | 2675 |
| L_cuneus_thickavg | 0.019 | 0.020 | -0.020 | 0.057 | 0.345 | 0.802 | 2538 |
| L_entorhinal_thickavg | 0.002 | 0.020 | -0.037 | 0.041 | 0.905 | 0.979 | 2510 |
| L_fusiform_thickavg | -0.010 | 0.019 | -0.048 | 0.028 | 0.595 | 0.971 | 2640 |
| L_inferiorparietal_thickavg | -0.006 | 0.020 | -0.044 | 0.033 | 0.776 | 0.973 | 2586 |
| L_inferiortemporal_thickavg | 0.004 | 0.020 | -0.035 | 0.042 | 0.855 | 0.979 | 2611 |
| L_isthmuscingulate_thickavg | -0.013 | 0.033 | -0.078 | 0.051 | 0.682 | 0.973 | 2686 |
| L_lateraloccipital_thickavg | -0.006 | 0.024 | -0.053 | 0.040 | 0.786 | 0.973 | 2644 |
| L_lateralorbitofrontal_thickavg | 0.018 | 0.028 | -0.038 | 0.073 | 0.526 | 0.971 | 2659 |
| L_lingual_thickavg | -0.007 | 0.019 | -0.045 | 0.031 | 0.704 | 0.973 | 2622 |
| L_medialorbitofrontal_thickavg | 0.049 | 0.021 | 0.008 | 0.090 | 0.020 | 0.247 | 2642 |
| L_middletemporal_thickavg | 0.001 | 0.020 | -0.038 | 0.041 | 0.940 | 0.979 | 2506 |
| L_parahippocampal_thickavg | 0.030 | 0.019 | -0.007 | 0.068 | 0.113 | 0.560 | 2671 |
| L_paracentral_thickavg | -0.017 | 0.019 | -0.054 | 0.021 | 0.387 | 0.861 | 2682 |
| L_parsopercularis_thickavg | 0.000 | 0.019 | -0.038 | 0.038 | 0.993 | 0.994 | 2658 |
| L_parsorbitalis_thickavg | 0.019 | 0.019 | -0.019 | 0.057 | 0.335 | 0.797 | 2664 |
| L_parstriangularis_thickavg | 0.019 | 0.025 | -0.031 | 0.069 | 0.460 | 0.925 | 2649 |
| L_pericalcarine_thickavg | 0.019 | 0.030 | -0.039 | 0.078 | 0.522 | 0.971 | 2592 |
| L_postcentral_thickavg | 0.009 | 0.025 | -0.040 | 0.057 | 0.729 | 0.973 | 2618 |
| L_posteriorcingulate_thickavg | 0.011 | 0.027 | -0.041 | 0.064 | 0.676 | 0.973 | 2685 |
| L_precentral_thickavg | 0.002 | 0.021 | -0.040 | 0.044 | 0.921 | 0.979 | 2638 |
| L_precuneus_thickavg | 0.002 | 0.021 | -0.040 | 0.044 | 0.935 | 0.979 | 2666 |
| L_rostralanteriorcingulate_thickavg | 0.060 | 0.019 | 0.022 | 0.098 | 0.002 | 0.036 | 2657 |
| L_rostralmiddlefrontal_thickavg | 0.006 | 0.031 | -0.055 | 0.067 | 0.855 | 0.979 | 2665 |
| L_superiorfrontal_thickavg | 0.029 | 0.025 | -0.019 | 0.077 | 0.242 | 0.780 | 2665 |
| L_superiorparietal_thickavg | 0.008 | 0.023 | -0.037 | 0.053 | 0.734 | 0.973 | 2623 |
| L_superiortemporal_thickavg | 0.011 | 0.020 | -0.028 | 0.050 | 0.573 | 0.971 | 2492 |
| L_supramarginal_thickavg | 0.004 | 0.020 | -0.036 | 0.044 | 0.862 | 0.979 | 2530 |
| L_frontalpole_thickavg | 0.049 | 0.019 | 0.012 | 0.087 | 0.010 | 0.168 | 2691 |
| L_temporalpole_thickavg | 0.031 | 0.019 | -0.007 | 0.069 | 0.105 | 0.560 | 2640 |
| L_transversetemporal_thickavg | 0.030 | 0.019 | -0.007 | 0.068 | 0.114 | 0.560 | 2691 |
| L_insula_thickavg | 0.025 | 0.025 | -0.024 | 0.074 | 0.316 | 0.797 | 2618 |
| R_bankssts_thickavg | -0.010 | 0.019 | -0.048 | 0.028 | 0.592 | 0.971 | 2620 |
| R_caudalanteriorcingulate_thickavg | 0.051 | 0.024 | 0.004 | 0.098 | 0.035 | 0.319 | 2683 |
| R_caudalmiddlefrontal_thickavg | 0.053 | 0.030 | -0.007 | 0.112 | 0.082 | 0.491 | 2667 |
| R_cuneus_thickavg | 0.018 | 0.024 | -0.029 | 0.065 | 0.456 | 0.925 | 2569 |
| R_entorhinal_thickavg | 0.029 | 0.023 | -0.017 | 0.075 | 0.211 | 0.763 | 2431 |
| R_fusiform_thickavg | 0.015 | 0.019 | -0.023 | 0.053 | 0.442 | 0.925 | 2647 |
| R_inferiorparietal_thickavg | 0.010 | 0.020 | -0.028 | 0.049 | 0.599 | 0.971 | 2584 |
| R_inferiortemporal_thickavg | 0.007 | 0.028 | -0.048 | 0.063 | 0.797 | 0.973 | 2621 |
| R_isthmuscingulate_thickavg | -0.012 | 0.028 | -0.067 | 0.043 | 0.669 | 0.973 | 2675 |
| R_lateraloccipital_thickavg | 0.000 | 0.019 | -0.038 | 0.038 | 0.994 | 0.994 | 2638 |
| R_lateralorbitofrontal_thickavg | 0.024 | 0.023 | -0.021 | 0.068 | 0.296 | 0.797 | 2636 |
| R_lingual_thickavg | 0.024 | 0.019 | -0.015 | 0.062 | 0.225 | 0.775 | 2623 |
| R_medialorbitofrontal_thickavg | 0.073 | 0.020 | 0.033 | 0.112 | 0.000 | 0.016 | 2622 |
| R_middletemporal_thickavg | 0.017 | 0.023 | -0.028 | 0.062 | 0.454 | 0.925 | 2593 |
| R_parahippocampal_thickavg | 0.008 | 0.019 | -0.030 | 0.046 | 0.686 | 0.973 | 2673 |
| R_paracentral_thickavg | 0.008 | 0.023 | -0.038 | 0.053 | 0.741 | 0.973 | 2679 |
| R_parsopercularis_thickavg | 0.039 | 0.019 | 0.001 | 0.077 | 0.044 | 0.370 | 2637 |
| R_parsorbitalis_thickavg | 0.037 | 0.037 | -0.036 | 0.111 | 0.323 | 0.797 | 2662 |
| R_parstriangularis_thickavg | 0.040 | 0.026 | -0.010 | 0.090 | 0.119 | 0.560 | 2628 |
| R_pericalcarine_thickavg | 0.051 | 0.022 | 0.008 | 0.093 | 0.020 | 0.247 | 2576 |
| R_postcentral_thickavg | -0.010 | 0.019 | -0.048 | 0.028 | 0.612 | 0.971 | 2632 |
| R_posteriorcingulate_thickavg | 0.038 | 0.027 | -0.015 | 0.090 | 0.157 | 0.685 | 2686 |
| R_precentral_thickavg | 0.018 | 0.034 | -0.049 | 0.085 | 0.592 | 0.971 | 2636 |
| R_precuneus_thickavg | -0.001 | 0.027 | -0.055 | 0.052 | 0.957 | 0.987 | 2670 |
| R_rostralanteriorcingulate_thickavg | 0.061 | 0.019 | 0.023 | 0.099 | 0.002 | 0.036 | 2633 |
| R_rostralmiddlefrontal_thickavg | 0.008 | 0.033 | -0.056 | 0.072 | 0.811 | 0.975 | 2643 |
| R_superiorfrontal_thickavg | 0.058 | 0.026 | 0.007 | 0.110 | 0.026 | 0.264 | 2664 |
| R_superiorparietal_thickavg | 0.020 | 0.020 | -0.020 | 0.059 | 0.328 | 0.797 | 2638 |
| R_superiortemporal_thickavg | 0.024 | 0.020 | -0.014 | 0.063 | 0.212 | 0.763 | 2569 |
| R_supramarginal_thickavg | 0.021 | 0.020 | -0.017 | 0.060 | 0.276 | 0.784 | 2559 |
| R_frontalpole_thickavg | 0.050 | 0.026 | -0.002 | 0.102 | 0.059 | 0.453 | 2682 |
| R_temporalpole_thickavg | 0.052 | 0.023 | 0.007 | 0.096 | 0.022 | 0.247 | 2577 |
| R_transversetemporal_thickavg | 0.015 | 0.024 | -0.032 | 0.062 | 0.523 | 0.971 | 2694 |
| R_insula_thickavg | 0.012 | 0.020 | -0.027 | 0.050 | 0.558 | 0.971 | 2585 |

Table S7. Positive schizotypy questionnaire moderator of association between CT and positive schizotypy

| DKT Regions | Q | p-value | FDR p-value |
| --- | --- | --- | --- |
| L_bankssts_thickavg | 14.274 | 0.075 | 0.259 |
| L_caudalanteriorcingulate_thickavg | 13.430 | 0.098 | 0.273 |
| L_caudalmiddlefrontal_thickavg | 17.828 | 0.023 | 0.125 |
| L_cuneus_thickavg | 9.784 | 0.281 | 0.481 |
| L_entorhinal_thickavg | 13.754 | 0.088 | 0.273 |
| L_frontalpole_thickavg | 11.621 | 0.169 | 0.392 |
| L_fusiform_thickavg | 10.545 | 0.229 | 0.438 |
| L_inferiorparietal_thickavg | 17.155 | 0.029 | 0.137 |
| L_inferiortemporal_thickavg | 10.348 | 0.241 | 0.438 |
| L_insula_thickavg | 11.514 | 0.174 | 0.392 |
| L_isthmuscingulate_thickavg | 13.411 | 0.098 | 0.273 |
| L_lateraloccipital_thickavg | 8.232 | 0.411 | 0.617 |
| L_lateralorbitofrontal_thickavg | 3.732 | 0.880 | 0.946 |
| L_lingual_thickavg | 10.051 | 0.261 | 0.459 |
| L_medialorbitofrontal_thickavg | 5.702 | 0.681 | 0.860 |
| L_middletemporal_thickavg | 11.804 | 0.160 | 0.384 |
| L_paracentral_thickavg | 5.004 | 0.757 | 0.879 |
| L_parahippocampal_thickavg | 2.348 | 0.968 | 0.982 |
| L_parsopercularis_thickavg | 15.693 | 0.047 | 0.188 |
| L_parsorbitalis_thickavg | 21.847 | 0.005 | 0.054 |
| L_parstriangularis_thickavg | 5.741 | 0.676 | 0.860 |
| L_pericalcarine_thickavg | 8.263 | 0.408 | 0.617 |
| L_postcentral_thickavg | 7.647 | 0.469 | 0.662 |
| L_posteriorcingulate_thickavg | 3.125 | 0.926 | 0.980 |
| L_precentral_thickavg | 23.180 | 0.003 | 0.040 |
| L_precuneus_thickavg | 31.255 | 0.000 | 0.005 |
| L_rostralanteriorcingulate_thickavg | 2.489 | 0.962 | 0.982 |
| L_rostralmiddlefrontal_thickavg | 14.245 | 0.076 | 0.259 |
| L_superiorfrontal_thickavg | 8.641 | 0.373 | 0.585 |
| L_superiorparietal_thickavg | 13.252 | 0.103 | 0.276 |
| L_superiortemporal_thickavg | 18.336 | 0.019 | 0.125 |
| L_supramarginal_thickavg | 10.316 | 0.244 | 0.438 |
| L_temporalpole_thickavg | 5.020 | 0.755 | 0.879 |
| L_transversetemporal_thickavg | 10.501 | 0.232 | 0.438 |
| R_bankssts_thickavg | 23.836 | 0.002 | 0.040 |
| R_caudalanteriorcingulate_thickavg | 18.075 | 0.021 | 0.125 |
| R_caudalmiddlefrontal_thickavg | 11.180 | 0.192 | 0.406 |
| R_cuneus_thickavg | 23.021 | 0.003 | 0.040 |
| R_entorhinal_thickavg | 1.727 | 0.988 | 0.988 |
| R_frontalpole_thickavg | 8.109 | 0.423 | 0.621 |
| R_fusiform_thickavg | 15.310 | 0.053 | 0.202 |
| R_inferiorparietal_thickavg | 15.995 | 0.042 | 0.188 |
| R_inferiortemporal_thickavg | 4.836 | 0.775 | 0.886 |
| R_insula_thickavg | 8.022 | 0.431 | 0.621 |
| R_isthmuscingulate_thickavg | 7.150 | 0.521 | 0.707 |
| R_lateraloccipital_thickavg | 4.379 | 0.821 | 0.924 |
| R_lateralorbitofrontal_thickavg | 5.085 | 0.748 | 0.879 |
| R_lingual_thickavg | 5.913 | 0.657 | 0.860 |
| R_medialorbitofrontal_thickavg | 10.658 | 0.222 | 0.438 |
| R_middletemporal_thickavg | 15.739 | 0.046 | 0.188 |
| R_paracentral_thickavg | 17.958 | 0.022 | 0.125 |
| R_parahippocampal_thickavg | 2.921 | 0.939 | 0.980 |
| R_parsopercularis_thickavg | 13.451 | 0.097 | 0.273 |
| R_parsorbitalis_thickavg | 6.323 | 0.611 | 0.815 |
| R_parstriangularis_thickavg | 4.241 | 0.835 | 0.925 |
| R_pericalcarine_thickavg | 10.713 | 0.218 | 0.438 |
| R_postcentral_thickavg | 5.304 | 0.725 | 0.879 |
| R_posteriorcingulate_thickavg | 7.336 | 0.501 | 0.693 |
| R_precentral_thickavg | 21.009 | 0.007 | 0.064 |
| R_precuneus_thickavg | 12.548 | 0.128 | 0.330 |
| R_rostralanteriorcingulate_thickavg | 5.348 | 0.720 | 0.879 |
| R_rostralmiddlefrontal_thickavg | 9.030 | 0.340 | 0.546 |
| R_superiorfrontal_thickavg | 27.783 | 0.001 | 0.012 |
| R_superiorparietal_thickavg | 20.318 | 0.009 | 0.074 |
| R_superiortemporal_thickavg | 32.057 | 0.000 | 0.005 |
| R_supramarginal_thickavg | 9.570 | 0.296 | 0.496 |
| R_temporalpole_thickavg | 9.012 | 0.341 | 0.546 |
| R_transversetemporal_thickavg | 3.763 | 0.878 | 0.946 |

# Table S8. Freesurfer version moderator of association with CT and positive schizotypy

| DKT regions | Q | p-value | FDR p-value |
| --- | --- | --- | --- |
| L_bankssts_thickavg | 0.902 | 0.970 | 0.953 |
| L_caudalanteriorcingulate_thickavg | 0.233 | 0.999 | 0.995 |
| L_caudalmiddlefrontal_thickavg | 2.346 | 0.800 | 0.953 |
| L_cuneus_thickavg | 4.581 | 0.469 | 0.953 |
| L_entorhinal_thickavg | 1.007 | 0.962 | 0.953 |
| L_fusiform_thickavg | 3.572 | 0.613 | 0.995 |
| L_inferiorparietal_thickavg | 3.096 | 0.685 | 0.953 |
| L_inferiortemporal_thickavg | 2.554 | 0.768 | 0.953 |
| L_isthmuscingulate_thickavg | 4.024 | 0.546 | 0.953 |
| L_lateraloccipital_thickavg | 2.991 | 0.701 | 0.953 |
| L_lateralorbitofrontal_thickavg | 4.232 | 0.516 | 0.953 |
| L_lingual_thickavg | 1.975 | 0.853 | 0.953 |
| L_medialorbitofrontal_thickavg | 9.625 | 0.087 | 0.953 |
| L_middletemporal_thickavg | 0.875 | 0.972 | 0.953 |
| L_parahippocampal_thickavg | 4.837 | 0.436 | 0.953 |
| L_paracentral_thickavg | 1.975 | 0.853 | 0.953 |
| L_parsopercularis_thickavg | 1.350 | 0.930 | 0.953 |
| L_parsorbitalis_thickavg | 8.423 | 0.134 | 0.953 |
| L_parstriangularis_thickavg | 1.307 | 0.934 | 0.953 |
| L_pericalcarine_thickavg | 4.119 | 0.532 | 0.953 |
| L_postcentral_thickavg | 8.864 | 0.115 | 0.953 |
| L_posteriorcingulate_thickavg | 3.713 | 0.591 | 0.953 |
| L_precentral_thickavg | 8.168 | 0.147 | 0.953 |
| L_precuneus_thickavg | 0.900 | 0.970 | 0.998 |
| L_rostralanteriorcingulate_thickavg | 4.092 | 0.536 | 0.953 |
| L_rostralmiddlefrontal_thickavg | 7.183 | 0.207 | 0.953 |
| L_superiorfrontal_thickavg | 2.640 | 0.755 | 0.953 |
| L_superiorparietal_thickavg | 2.814 | 0.729 | 0.953 |
| L_superiortemporal_thickavg | 3.170 | 0.674 | 0.953 |
| L_supramarginal_thickavg | 4.129 | 0.531 | 0.995 |
| L_frontalpole_thickavg | 3.285 | 0.656 | 0.995 |
| L_temporalpole_thickavg | 2.911 | 0.714 | 0.953 |
| L_transversetemporal_thickavg | 5.417 | 0.367 | 0.953 |
| L_insula_thickavg | 5.981 | 0.308 | 0.953 |
| R_bankssts_thickavg | 5.013 | 0.414 | 0.953 |
| R_caudalanteriorcingulate_thickavg | 5.375 | 0.372 | 0.953 |
| R_caudalmiddlefrontal_thickavg | 8.295 | 0.141 | 0.953 |
| R_cuneus_thickavg | 4.351 | 0.500 | 0.953 |
| R_entorhinal_thickavg | 2.333 | 0.801 | 0.953 |
| R_fusiform_thickavg | 3.421 | 0.635 | 0.953 |
| R_inferiorparietal_thickavg | 2.721 | 0.743 | 0.953 |
| R_inferiortemporal_thickavg | 1.827 | 0.872 | 0.953 |
| R_isthmuscingulate_thickavg | 7.385 | 0.194 | 0.953 |
| R_lateraloccipital_thickavg | 5.431 | 0.366 | 0.953 |
| R_lateralorbitofrontal_thickavg | 4.561 | 0.472 | 0.953 |
| R_lingual_thickavg | 6.731 | 0.241 | 0.953 |
| R_medialorbitofrontal_thickavg | 14.690 | 0.012 | 0.953 |
| R_middletemporal_thickavg | 1.121 | 0.952 | 0.953 |
| R_parahippocampal_thickavg | 5.833 | 0.323 | 0.953 |
| R_paracentral_thickavg | 0.558 | 0.990 | 0.995 |
| R_parsopercularis_thickavg | 10.106 | 0.072 | 0.953 |
| R_parsorbitalis_thickavg | 4.084 | 0.537 | 0.953 |
| R_parstriangularis_thickavg | 7.357 | 0.195 | 0.953 |
| R_pericalcarine_thickavg | 9.595 | 0.088 | 0.953 |
| R_postcentral_thickavg | 6.304 | 0.278 | 0.953 |
| R_posteriorcingulate_thickavg | 2.524 | 0.773 | 0.953 |
| R_precentral_thickavg | 4.723 | 0.451 | 0.953 |
| R_precuneus_thickavg | 2.063 | 0.840 | 0.953 |
| R_rostralanteriorcingulate_thickavg | 5.578 | 0.349 | 0.953 |
| R_rostralmiddlefrontal_thickavg | 1.912 | 0.861 | 0.953 |
| R_superiorfrontal_thickavg | 7.094 | 0.214 | 0.953 |
| R_superiorparietal_thickavg | 3.296 | 0.654 | 0.953 |
| R_superiortemporal_thickavg | 3.755 | 0.585 | 0.953 |
| R_supramarginal_thickavg | 3.381 | 0.641 | 0.953 |
| R_frontalpole_thickavg | 3.846 | 0.572 | 0.953 |
| R_temporalpole_thickavg | 5.780 | 0.328 | 0.998 |
| R_transversetemporal_thickavg | 5.900 | 0.316 | 0.953 |
| R_insula_thickavg | 0.600 | 0.988 | 0.953 |

# Table S9. Field strength moderator of association with CT and positive schizotypy

| DKT regions | Q | p-value | FDR-adjusted p-value |
| --- | --- | --- | --- |
| L_bankssts_thickavg | 0.791 | 0.673 | 0.953 |
| L_caudalanteriorcingulate_thickavg | 0.115 | 0.944 | 0.995 |
| L_caudalmiddlefrontal_thickavg | 0.392 | 0.822 | 0.953 |
| L_cuneus_thickavg | 0.425 | 0.809 | 0.953 |
| L_entorhinal_thickavg | 0.911 | 0.634 | 0.953 |
| L_fusiform_thickavg | 0.184 | 0.912 | 0.995 |
| L_inferiorparietal_thickavg | 0.452 | 0.798 | 0.953 |
| L_inferiortemporal_thickavg | 1.426 | 0.490 | 0.953 |
| L_isthmuscingulate_thickavg | 1.734 | 0.420 | 0.953 |
| L_lateraloccipital_thickavg | 0.997 | 0.607 | 0.953 |
| L_lateralorbitofrontal_thickavg | 2.957 | 0.228 | 0.953 |
| L_lingual_thickavg | 0.943 | 0.624 | 0.953 |
| L_medialorbitofrontal_thickavg | 4.074 | 0.130 | 0.953 |
| L_middletemporal_thickavg | 0.337 | 0.845 | 0.953 |
| L_parahippocampal_thickavg | 3.831 | 0.147 | 0.953 |
| L_paracentral_thickavg | 1.638 | 0.441 | 0.953 |
| L_parsopercularis_thickavg | 0.640 | 0.726 | 0.953 |
| L_parsorbitalis_thickavg | 1.184 | 0.553 | 0.953 |
| L_parstriangularis_thickavg | 0.605 | 0.739 | 0.953 |
| L_pericalcarine_thickavg | 0.812 | 0.666 | 0.953 |
| L_postcentral_thickavg | 0.345 | 0.842 | 0.953 |
| L_posteriorcingulate_thickavg | 0.377 | 0.828 | 0.953 |
| L_precentral_thickavg | 1.370 | 0.504 | 0.953 |
| L_precuneus_thickavg | 0.004 | 0.998 | 0.998 |
| L_rostralanteriorcingulate_thickavg | 0.474 | 0.789 | 0.953 |
| L_rostralmiddlefrontal_thickavg | 3.532 | 0.171 | 0.953 |
| L_superiorfrontal_thickavg | 2.313 | 0.315 | 0.953 |
| L_superiorparietal_thickavg | 0.693 | 0.707 | 0.953 |
| L_superiortemporal_thickavg | 0.563 | 0.755 | 0.953 |
| L_supramarginal_thickavg | 0.071 | 0.965 | 0.995 |
| L_frontalpole_thickavg | 0.112 | 0.945 | 0.995 |
| L_temporalpole_thickavg | 0.797 | 0.671 | 0.953 |
| L_transversetemporal_thickavg | 5.257 | 0.072 | 0.953 |
| L_insula_thickavg | 3.155 | 0.206 | 0.953 |
| R_bankssts_thickavg | 4.260 | 0.119 | 0.953 |
| R_caudalanteriorcingulate_thickavg | 2.505 | 0.286 | 0.953 |
| R_caudalmiddlefrontal_thickavg | 3.453 | 0.178 | 0.953 |
| R_cuneus_thickavg | 0.836 | 0.658 | 0.953 |
| R_entorhinal_thickavg | 1.360 | 0.507 | 0.953 |
| R_fusiform_thickavg | 0.570 | 0.752 | 0.953 |
| R_inferiorparietal_thickavg | 2.466 | 0.291 | 0.953 |
| R_inferiortemporal_thickavg | 0.320 | 0.852 | 0.953 |
| R_isthmuscingulate_thickavg | 1.359 | 0.507 | 0.953 |
| R_lateraloccipital_thickavg | 0.305 | 0.859 | 0.953 |
| R_lateralorbitofrontal_thickavg | 4.524 | 0.104 | 0.953 |
| R_lingual_thickavg | 1.179 | 0.555 | 0.953 |
| R_medialorbitofrontal_thickavg | 8.611 | 0.013 | 0.953 |
| R_middletemporal_thickavg | 0.431 | 0.806 | 0.953 |
| R_parahippocampal_thickavg | 0.969 | 0.616 | 0.953 |
| R_paracentral_thickavg | 0.066 | 0.968 | 0.995 |
| R_parsopercularis_thickavg | 1.533 | 0.465 | 0.953 |
| R_parsorbitalis_thickavg | 2.394 | 0.302 | 0.953 |
| R_parstriangularis_thickavg | 2.215 | 0.330 | 0.953 |
| R_pericalcarine_thickavg | 1.530 | 0.465 | 0.953 |
| R_postcentral_thickavg | 1.467 | 0.480 | 0.953 |
| R_posteriorcingulate_thickavg | 0.610 | 0.737 | 0.953 |
| R_precentral_thickavg | 1.270 | 0.530 | 0.953 |
| R_precuneus_thickavg | 0.832 | 0.660 | 0.953 |
| R_rostralanteriorcingulate_thickavg | 1.626 | 0.444 | 0.953 |
| R_rostralmiddlefrontal_thickavg | 1.581 | 0.454 | 0.953 |
| R_superiorfrontal_thickavg | 2.459 | 0.292 | 0.953 |
| R_superiorparietal_thickavg | 1.536 | 0.464 | 0.953 |
| R_superiortemporal_thickavg | 0.664 | 0.718 | 0.953 |
| R_supramarginal_thickavg | 0.691 | 0.708 | 0.953 |
| R_frontalpole_thickavg | 2.521 | 0.283 | 0.953 |
| R_temporalpole_thickavg | 0.009 | 0.996 | 0.998 |
| R_transversetemporal_thickavg | 2.030 | 0.362 | 0.953 |
| R_insula_thickavg | 0.367 | 0.832 | 0.953 |

# Table S10. Negative schizotypy questionnaire moderator CT and negative schizotypy association

| DKT regions | Q | p-value | FDR p-value |
| --- | --- | --- | --- |
| L_bankssts_thickavg | 6.612 | 0.251 | 0.887 |
| L_caudalanteriorcingulate_thickavg | 1.509 | 0.912 | 0.999 |
| L_caudalmiddlefrontal_thickavg | 6.398 | 0.269 | 0.887 |
| L_cuneus_thickavg | 7.819 | 0.166 | 0.821 |
| L_entorhinal_thickavg | 4.980 | 0.418 | 0.911 |
| L_frontalpole_thickavg | 2.481 | 0.779 | 0.960 |
| L_fusiform_thickavg | 3.475 | 0.627 | 0.911 |
| L_inferiorparietal_thickavg | 2.398 | 0.792 | 0.960 |
| L_inferiortemporal_thickavg | 4.143 | 0.529 | 0.911 |
| L_insula_thickavg | 6.205 | 0.287 | 0.887 |
| L_isthmuscingulate_thickavg | 0.532 | 0.991 | 0.999 |
| L_lateraloccipital_thickavg | 2.976 | 0.704 | 0.911 |
| L_lateralorbitofrontal_thickavg | 9.222 | 0.101 | 0.531 |
| L_lingual_thickavg | 1.200 | 0.945 | 0.999 |
| L_medialorbitofrontal_thickavg | 2.910 | 0.714 | 0.911 |
| L_middletemporal_thickavg | 1.467 | 0.917 | 0.999 |
| L_paracentral_thickavg | 3.495 | 0.624 | 0.911 |
| L_parahippocampal_thickavg | 3.096 | 0.685 | 0.911 |
| L_parsopercularis_thickavg | 13.511 | 0.019 | 0.282 |
| L_parsorbitalis_thickavg | 4.165 | 0.526 | 0.911 |
| L_parstriangularis_thickavg | 1.457 | 0.918 | 0.999 |
| L_pericalcarine_thickavg | 1.055 | 0.958 | 0.999 |
| L_postcentral_thickavg | 6.006 | 0.306 | 0.887 |
| L_posteriorcingulate_thickavg | 3.423 | 0.635 | 0.911 |
| L_precentral_thickavg | 5.380 | 0.371 | 0.887 |
| L_precuneus_thickavg | 3.672 | 0.597 | 0.911 |
| L_rostralanteriorcingulate_thickavg | 4.517 | 0.478 | 0.911 |
| L_rostralmiddlefrontal_thickavg | 5.189 | 0.393 | 0.909 |
| L_superiorfrontal_thickavg | 5.542 | 0.353 | 0.887 |
| L_superiorparietal_thickavg | 1.755 | 0.882 | 0.999 |
| L_superiortemporal_thickavg | 5.570 | 0.350 | 0.887 |
| L_supramarginal_thickavg | 5.920 | 0.314 | 0.887 |
| L_temporalpole_thickavg | 4.253 | 0.514 | 0.911 |
| L_transversetemporal_thickavg | 2.506 | 0.776 | 0.960 |
| R_bankssts_thickavg | 11.634 | 0.040 | 0.495 |
| R_caudalanteriorcingulate_thickavg | 5.378 | 0.371 | 0.887 |
| R_caudalmiddlefrontal_thickavg | 23.504 | 0.000 | 0.020 |
| R_cuneus_thickavg | 0.993 | 0.963 | 0.999 |
| R_entorhinal_thickavg | 3.527 | 0.619 | 0.911 |
| R_frontalpole_thickavg | 6.075 | 0.299 | 0.887 |
| R_fusiform_thickavg | 3.224 | 0.665 | 0.911 |
| R_inferiorparietal_thickavg | 3.794 | 0.579 | 0.911 |
| R_inferiortemporal_thickavg | 9.993 | 0.075 | 0.529 |
| R_insula_thickavg | 7.015 | 0.220 | 0.887 |
| R_isthmuscingulate_thickavg | 1.847 | 0.870 | 0.999 |
| R_lateraloccipital_thickavg | 2.987 | 0.702 | 0.911 |
| R_lateralorbitofrontal_thickavg | 4.251 | 0.514 | 0.911 |
| R_lingual_thickavg | 3.273 | 0.658 | 0.911 |
| R_medialorbitofrontal_thickavg | 5.598 | 0.347 | 0.887 |
| R_middletemporal_thickavg | 6.221 | 0.285 | 0.887 |
| R_paracentral_thickavg | 18.231 | 0.003 | 0.099 |
| R_parahippocampal_thickavg | 6.295 | 0.279 | 0.887 |
| R_parsopercularis_thickavg | 9.438 | 0.093 | 0.529 |
| R_parsorbitalis_thickavg | 9.503 | 0.091 | 0.529 |
| R_parstriangularis_thickavg | 13.697 | 0.018 | 0.282 |
| R_pericalcarine_thickavg | 4.029 | 0.545 | 0.911 |
| R_postcentral_thickavg | 3.464 | 0.629 | 0.911 |
| R_posteriorcingulate_thickavg | 3.699 | 0.593 | 0.911 |
| R_precentral_thickavg | 3.274 | 0.658 | 0.911 |
| R_precuneus_thickavg | 2.163 | 0.826 | 0.986 |
| R_rostralanteriorcingulate_thickavg | 5.462 | 0.362 | 0.887 |
| R_rostralmiddlefrontal_thickavg | 10.238 | 0.069 | 0.529 |
| R_superiorfrontal_thickavg | 14.554 | 0.012 | 0.282 |
| R_superiorparietal_thickavg | 4.806 | 0.440 | 0.911 |
| R_superiortemporal_thickavg | 10.210 | 0.069 | 0.529 |
| R_supramarginal_thickavg | 3.928 | 0.560 | 0.911 |
| R_temporalpole_thickavg | 3.894 | 0.565 | 0.911 |
| R_transversetemporal_thickavg | 0.792 | 0.978 | 0.999 |

# Table S11. Freesurfer version moderator of association with CT and negative schizotypy

| DTK regions | Q | p-value | FDR p-value |
| --- | --- | --- | --- |
| L_bankssts_thickavg | 5.525 | 0.355 | 0.822 |
| L_caudalanteriorcingulate_thickavg | 3.440 | 0.632 | 0.947 |
| L_caudalmiddlefrontal_thickavg | 2.654 | 0.753 | 0.947 |
| L_cuneus_thickavg | 9.123 | 0.104 | 0.563 |
| L_entorhinal_thickavg | 4.287 | 0.509 | 0.897 |
| L_frontalpole_thickavg | 8.586 | 0.127 | 0.563 |
| L_fusiform_thickavg | 6.372 | 0.272 | 0.649 |
| L_inferiorparietal_thickavg | 2.674 | 0.750 | 0.947 |
| L_inferiortemporal_thickavg | 8.804 | 0.117 | 0.563 |
| L_insula_thickavg | 11.641 | 0.040 | 0.563 |
| L_isthmuscingulate_thickavg | 0.220 | 0.999 | 0.999 |
| L_lateraloccipital_thickavg | 3.066 | 0.690 | 0.947 |
| L_lateralorbitofrontal_thickavg | 21.071 | 0.001 | 0.058 |
| L_lingual_thickavg | 3.380 | 0.642 | 0.947 |
| L_medialorbitofrontal_thickavg | 4.928 | 0.425 | 0.873 |
| L_middletemporal_thickavg | 6.778 | 0.238 | 0.648 |
| L_paracentral_thickavg | 2.533 | 0.772 | 0.947 |
| L_parahippocampal_thickavg | 9.754 | 0.083 | 0.563 |
| L_parsopercularis_thickavg | 9.926 | 0.077 | 0.563 |
| L_parsorbitalis_thickavg | 2.967 | 0.705 | 0.947 |
| L_parstriangularis_thickavg | 3.945 | 0.557 | 0.937 |
| L_pericalcarine_thickavg | 3.158 | 0.676 | 0.947 |
| L_postcentral_thickavg | 8.676 | 0.123 | 0.563 |
| L_posteriorcingulate_thickavg | 8.043 | 0.154 | 0.592 |
| L_precentral_thickavg | 2.050 | 0.842 | 0.999 |
| L_precuneus_thickavg | 3.208 | 0.668 | 0.947 |
| L_rostralanteriorcingulate_thickavg | 7.590 | 0.180 | 0.599 |
| L_rostralmiddlefrontal_thickavg | 4.335 | 0.502 | 0.897 |
| L_superiorfrontal_thickavg | 2.834 | 0.726 | 0.947 |
| L_superiorparietal_thickavg | 2.876 | 0.719 | 0.947 |
| L_superiortemporal_thickavg | 4.432 | 0.489 | 0.897 |
| L_supramarginal_thickavg | 7.589 | 0.180 | 0.599 |
| L_temporalpole_thickavg | 6.391 | 0.270 | 0.649 |
| L_transversetemporal_thickavg | 2.725 | 0.742 | 0.947 |
| R_bankssts_thickavg | 10.139 | 0.071 | 0.563 |
| R_caudalanteriorcingulate_thickavg | 4.738 | 0.449 | 0.876 |
| R_caudalmiddlefrontal_thickavg | 7.497 | 0.186 | 0.599 |
| R_cuneus_thickavg | 8.528 | 0.129 | 0.563 |
| R_entorhinal_thickavg | 3.073 | 0.689 | 0.947 |
| R_frontalpole_thickavg | 1.542 | 0.908 | 0.999 |
| R_fusiform_thickavg | 2.472 | 0.781 | 0.947 |
| R_inferiorparietal_thickavg | 1.521 | 0.911 | 0.999 |
| R_inferiortemporal_thickavg | 7.333 | 0.197 | 0.608 |
| R_insula_thickavg | 8.553 | 0.128 | 0.563 |
| R_isthmuscingulate_thickavg | 1.862 | 0.868 | 0.999 |
| R_lateraloccipital_thickavg | 4.729 | 0.450 | 0.876 |
| R_lateralorbitofrontal_thickavg | 7.149 | 0.210 | 0.621 |
| R_lingual_thickavg | 6.736 | 0.241 | 0.648 |
| R_medialorbitofrontal_thickavg | 10.734 | 0.057 | 0.563 |
| R_middletemporal_thickavg | 8.729 | 0.120 | 0.563 |
| R_paracentral_thickavg | 6.654 | 0.248 | 0.648 |
| R_parahippocampal_thickavg | 17.773 | 0.003 | 0.120 |
| R_parsopercularis_thickavg | 8.019 | 0.155 | 0.592 |
| R_parsorbitalis_thickavg | 2.638 | 0.756 | 0.947 |
| R_parstriangularis_thickavg | 14.229 | 0.014 | 0.351 |
| R_pericalcarine_thickavg | 0.993 | 0.963 | 0.999 |
| R_postcentral_thickavg | 4.974 | 0.419 | 0.873 |
| R_posteriorcingulate_thickavg | 1.118 | 0.953 | 0.999 |
| R_precentral_thickavg | 1.869 | 0.867 | 0.999 |
| R_precuneus_thickavg | 1.433 | 0.921 | 0.999 |
| R_rostralanteriorcingulate_thickavg | 0.517 | 0.992 | 0.999 |
| R_rostralmiddlefrontal_thickavg | 4.579 | 0.469 | 0.891 |
| R_superiorfrontal_thickavg | 3.263 | 0.659 | 0.947 |
| R_superiorparietal_thickavg | 0.906 | 0.970 | 0.999 |
| R_superiortemporal_thickavg | 6.579 | 0.254 | 0.648 |
| R_supramarginal_thickavg | 7.932 | 0.160 | 0.592 |
| R_temporalpole_thickavg | 10.450 | 0.063 | 0.563 |
| R_transversetemporal_thickavg | 8.578 | 0.127 | 0.563 |

# Table S12. Field strength moderator of association with CT and negative schizotypy

| DKT regions | Q | p-value | FDR p-value |
| --- | --- | --- | --- |
| L_bankssts_thickavg | 0.802 | 0.370 | 0.977 |
| L_caudalanteriorcingulate_thickavg | 0.439 | 0.508 | 0.977 |
| L_caudalmiddlefrontal_thickavg | 0.009 | 0.927 | 0.977 |
| L_cuneus_thickavg | 2.717 | 0.099 | 0.977 |
| L_entorhinal_thickavg | 0.163 | 0.686 | 0.977 |
| L_frontalpole_thickavg | 2.652 | 0.103 | 0.977 |
| L_fusiform_thickavg | 0.036 | 0.849 | 0.977 |
| L_inferiorparietal_thickavg | 0.819 | 0.365 | 0.977 |
| L_inferiortemporal_thickavg | 0.606 | 0.436 | 0.977 |
| L_insula_thickavg | 0.783 | 0.376 | 0.977 |
| L_isthmuscingulate_thickavg | 0.004 | 0.947 | 0.977 |
| L_lateraloccipital_thickavg | 0.228 | 0.633 | 0.977 |
| L_lateralorbitofrontal_thickavg | 0.007 | 0.934 | 0.977 |
| L_lingual_thickavg | 0.115 | 0.734 | 0.977 |
| L_medialorbitofrontal_thickavg | 0.124 | 0.725 | 0.977 |
| L_middletemporal_thickavg | 0.950 | 0.330 | 0.977 |
| L_paracentral_thickavg | 0.004 | 0.950 | 0.977 |
| L_parahippocampal_thickavg | 1.740 | 0.187 | 0.977 |
| L_parsopercularis_thickavg | 0.821 | 0.365 | 0.977 |
| L_parsorbitalis_thickavg | 0.795 | 0.373 | 0.977 |
| L_parstriangularis_thickavg | 0.094 | 0.759 | 0.977 |
| L_pericalcarine_thickavg | 0.541 | 0.462 | 0.977 |
| L_postcentral_thickavg | 0.565 | 0.452 | 0.977 |
| L_posteriorcingulate_thickavg | 0.031 | 0.860 | 0.977 |
| L_precentral_thickavg | 0.246 | 0.620 | 0.977 |
| L_precuneus_thickavg | 0.690 | 0.406 | 0.977 |
| L_rostralanteriorcingulate_thickavg | 1.229 | 0.268 | 0.977 |
| L_rostralmiddlefrontal_thickavg | 0.075 | 0.784 | 0.977 |
| L_superiorfrontal_thickavg | 0.028 | 0.867 | 0.977 |
| L_superiorparietal_thickavg | 0.005 | 0.944 | 0.977 |
| L_superiortemporal_thickavg | 0.337 | 0.562 | 0.977 |
| L_supramarginal_thickavg | 0.185 | 0.667 | 0.977 |
| L_temporalpole_thickavg | 0.297 | 0.586 | 0.977 |
| L_transversetemporal_thickavg | 0.307 | 0.579 | 0.977 |
| R_bankssts_thickavg | 3.749 | 0.053 | 0.977 |
| R_caudalanteriorcingulate_thickavg | 0.019 | 0.890 | 0.977 |
| R_caudalmiddlefrontal_thickavg | 0.033 | 0.857 | 0.977 |
| R_cuneus_thickavg | 0.273 | 0.601 | 0.977 |
| R_entorhinal_thickavg | 0.581 | 0.446 | 0.977 |
| R_frontalpole_thickavg | 0.279 | 0.597 | 0.977 |
| R_fusiform_thickavg | 0.031 | 0.860 | 0.977 |
| R_inferiorparietal_thickavg | 0.205 | 0.651 | 0.977 |
| R_inferiortemporal_thickavg | 1.763 | 0.184 | 0.977 |
| R_insula_thickavg | 1.756 | 0.185 | 0.977 |
| R_isthmuscingulate_thickavg | 0.210 | 0.647 | 0.977 |
| R_lateraloccipital_thickavg | 1.042 | 0.307 | 0.977 |
| R_lateralorbitofrontal_thickavg | 0.464 | 0.496 | 0.977 |
| R_lingual_thickavg | 0.152 | 0.696 | 0.977 |
| R_medialorbitofrontal_thickavg | 0.021 | 0.884 | 0.977 |
| R_middletemporal_thickavg | 0.128 | 0.720 | 0.977 |
| R_paracentral_thickavg | 0.033 | 0.855 | 0.977 |
| R_parahippocampal_thickavg | 9.874 | 0.002 | 0.124 |
| R_parsopercularis_thickavg | 1.683 | 0.194 | 0.977 |
| R_parsorbitalis_thickavg | 0.049 | 0.825 | 0.977 |
| R_parstriangularis_thickavg | 0.129 | 0.719 | 0.977 |
| R_pericalcarine_thickavg | 0.053 | 0.817 | 0.977 |
| R_postcentral_thickavg | 0.977 | 0.323 | 0.977 |
| R_posteriorcingulate_thickavg | 0.030 | 0.863 | 0.977 |
| R_precentral_thickavg | 0.008 | 0.928 | 0.977 |
| R_precuneus_thickavg | 0.000 | 0.997 | 0.997 |
| R_rostralanteriorcingulate_thickavg | 0.002 | 0.966 | 0.979 |
| R_rostralmiddlefrontal_thickavg | 0.149 | 0.700 | 0.977 |
| R_superiorfrontal_thickavg | 0.120 | 0.729 | 0.977 |
| R_superiorparietal_thickavg | 0.141 | 0.707 | 0.977 |
| R_superiortemporal_thickavg | 0.260 | 0.610 | 0.977 |
| R_supramarginal_thickavg | 0.681 | 0.409 | 0.977 |
| R_temporalpole_thickavg | 1.363 | 0.243 | 0.977 |
| R_transversetemporal_thickavg | 2.072 | 0.150 | 0.977 |

# Table S13. Association between SA and positive schizotypy corrected for age, and sex

| DKT Regions | Partial R | Std. Err. | 95% CI, lower bound | 95% CI, upper bound | p-value | FDR p-value | Sample size per region |
| --- | --- | --- | --- | --- | --- | --- | --- |
| L_bankssts_surfavg | 0.015 | 0.020 | -0.025 | 0.055 | 0.465 | 0.934 | 2394 |
| L_caudalanteriorcingulate_surfavg | -0.011 | 0.033 | -0.075 | 0.053 | 0.731 | 0.938 | 2554 |
| L_caudalmiddlefrontal_surfavg | -0.053 | 0.022 | -0.097 | -0.009 | 0.017 | 0.394 | 2559 |
| L_cuneus_surfavg | -0.041 | 0.029 | -0.097 | 0.016 | 0.157 | 0.836 | 2420 |
| L_entorhinal_surfavg | -0.036 | 0.020 | -0.075 | 0.004 | 0.081 | 0.622 | 2395 |
| L_fusiform_surfavg | -0.002 | 0.030 | -0.060 | 0.056 | 0.953 | 0.973 | 2526 |
| L_inferiorparietal_surfavg | 0.014 | 0.034 | -0.052 | 0.080 | 0.684 | 0.934 | 2470 |
| L_inferiortemporal_surfavg | 0.014 | 0.022 | -0.028 | 0.057 | 0.509 | 0.934 | 2496 |
| L_isthmuscingulate_surfavg | 0.007 | 0.027 | -0.045 | 0.059 | 0.778 | 0.938 | 2571 |
| L_lateraloccipital_surfavg | -0.029 | 0.031 | -0.090 | 0.032 | 0.356 | 0.934 | 2529 |
| L_lateralorbitofrontal_surfavg | -0.016 | 0.025 | -0.065 | 0.033 | 0.516 | 0.934 | 2544 |
| L_lingual_surfavg | 0.004 | 0.027 | -0.049 | 0.056 | 0.891 | 0.961 | 2506 |
| L_medialorbitofrontal_surfavg | -0.049 | 0.022 | -0.091 | -0.007 | 0.024 | 0.394 | 2527 |
| L_middletemporal_surfavg | 0.033 | 0.027 | -0.021 | 0.086 | 0.234 | 0.836 | 2388 |
| L_parahippocampal_surfavg | -0.043 | 0.020 | -0.082 | -0.005 | 0.027 | 0.394 | 2555 |
| L_paracentral_surfavg | -0.008 | 0.037 | -0.081 | 0.064 | 0.822 | 0.945 | 2567 |
| L_parsopercularis_surfavg | 0.026 | 0.022 | -0.016 | 0.069 | 0.224 | 0.836 | 2544 |
| L_parsorbitalis_surfavg | 0.003 | 0.030 | -0.056 | 0.062 | 0.930 | 0.961 | 2549 |
| L_parstriangularis_surfavg | 0.003 | 0.020 | -0.035 | 0.042 | 0.874 | 0.961 | 2535 |
| L_pericalcarine_surfavg | -0.021 | 0.024 | -0.068 | 0.025 | 0.368 | 0.934 | 2473 |
| L_postcentral_surfavg | -0.011 | 0.024 | -0.058 | 0.036 | 0.653 | 0.934 | 2505 |
| L_posteriorcingulate_surfavg | 0.017 | 0.021 | -0.024 | 0.058 | 0.411 | 0.934 | 2568 |
| L_precentral_surfavg | -0.031 | 0.023 | -0.077 | 0.015 | 0.187 | 0.836 | 2523 |
| L_precuneus_surfavg | -0.019 | 0.035 | -0.087 | 0.050 | 0.597 | 0.934 | 2558 |
| L_rostralanteriorcingulate_surfavg | -0.043 | 0.023 | -0.089 | 0.002 | 0.063 | 0.622 | 2542 |
| L_rostralmiddlefrontal_surfavg | -0.025 | 0.030 | -0.085 | 0.034 | 0.409 | 0.934 | 2550 |
| L_superiorfrontal_surfavg | -0.023 | 0.026 | -0.073 | 0.027 | 0.371 | 0.934 | 2550 |
| L_superiorparietal_surfavg | 0.013 | 0.030 | -0.046 | 0.071 | 0.668 | 0.934 | 2512 |
| L_superiortemporal_surfavg | -0.013 | 0.025 | -0.062 | 0.036 | 0.613 | 0.934 | 2378 |
| L_supramarginal_surfavg | 0.008 | 0.028 | -0.046 | 0.062 | 0.775 | 0.938 | 2418 |
| L_frontalpole_surfavg | -0.015 | 0.020 | -0.054 | 0.023 | 0.434 | 0.934 | 2576 |
| L_temporalpole_surfavg | -0.030 | 0.029 | -0.087 | 0.027 | 0.301 | 0.900 | 2522 |
| L_transversetemporal_surfavg | -0.023 | 0.022 | -0.065 | 0.020 | 0.292 | 0.900 | 2576 |
| L_insula_surfavg | -0.013 | 0.032 | -0.075 | 0.050 | 0.686 | 0.934 | 2498 |
| R_bankssts_surfavg | 0.017 | 0.026 | -0.034 | 0.067 | 0.521 | 0.934 | 2505 |
| R_caudalanteriorcingulate_surfavg | 0.000 | 0.022 | -0.044 | 0.044 | 0.996 | 0.996 | 2566 |
| R_caudalmiddlefrontal_surfavg | -0.026 | 0.020 | -0.066 | 0.013 | 0.191 | 0.836 | 2550 |
| R_cuneus_surfavg | -0.053 | 0.030 | -0.112 | 0.006 | 0.076 | 0.622 | 2449 |
| R_entorhinal_surfavg | -0.056 | 0.026 | -0.107 | -0.005 | 0.032 | 0.401 | 2309 |
| R_fusiform_surfavg | 0.024 | 0.021 | -0.018 | 0.066 | 0.265 | 0.856 | 2532 |
| R_inferiorparietal_surfavg | 0.022 | 0.031 | -0.038 | 0.083 | 0.468 | 0.934 | 2468 |
| R_inferiortemporal_surfavg | -0.003 | 0.023 | -0.048 | 0.043 | 0.903 | 0.961 | 2506 |
| R_isthmuscingulate_surfavg | 0.008 | 0.030 | -0.052 | 0.068 | 0.790 | 0.940 | 2560 |
| R_lateraloccipital_surfavg | -0.001 | 0.022 | -0.044 | 0.042 | 0.965 | 0.975 | 2522 |
| R_lateralorbitofrontal_surfavg | -0.025 | 0.020 | -0.064 | 0.014 | 0.209 | 0.836 | 2520 |
| R_lingual_surfavg | -0.011 | 0.020 | -0.050 | 0.028 | 0.565 | 0.934 | 2508 |
| R_medialorbitofrontal_surfavg | -0.033 | 0.020 | -0.072 | 0.006 | 0.099 | 0.663 | 2502 |
| R_middletemporal_surfavg | 0.005 | 0.027 | -0.048 | 0.058 | 0.851 | 0.956 | 2480 |
| R_parahippocampal_surfavg | 0.007 | 0.020 | -0.032 | 0.045 | 0.739 | 0.938 | 2558 |
| R_paracentral_surfavg | -0.026 | 0.033 | -0.092 | 0.039 | 0.429 | 0.934 | 2564 |
| R_parsopercularis_surfavg | 0.007 | 0.022 | -0.036 | 0.051 | 0.745 | 0.938 | 2519 |
| R_parsorbitalis_surfavg | -0.025 | 0.020 | -0.064 | 0.013 | 0.196 | 0.836 | 2547 |
| R_parstriangularis_surfavg | 0.010 | 0.024 | -0.036 | 0.056 | 0.666 | 0.934 | 2513 |
| R_pericalcarine_surfavg | -0.031 | 0.020 | -0.070 | 0.009 | 0.126 | 0.787 | 2459 |
| R_postcentral_surfavg | -0.011 | 0.028 | -0.065 | 0.043 | 0.701 | 0.934 | 2515 |
| R_posteriorcingulate_surfavg | -0.020 | 0.029 | -0.077 | 0.036 | 0.480 | 0.934 | 2569 |
| R_precentral_surfavg | -0.014 | 0.020 | -0.053 | 0.025 | 0.495 | 0.934 | 2517 |
| R_precuneus_surfavg | 0.003 | 0.031 | -0.057 | 0.064 | 0.914 | 0.961 | 2558 |
| R_rostralanteriorcingulate_surfavg | 0.019 | 0.025 | -0.030 | 0.068 | 0.456 | 0.934 | 2511 |
| R_rostralmiddlefrontal_surfavg | -0.003 | 0.028 | -0.059 | 0.052 | 0.907 | 0.961 | 2528 |
| R_superiorfrontal_surfavg | -0.034 | 0.029 | -0.091 | 0.022 | 0.234 | 0.836 | 2549 |
| R_superiorparietal_surfavg | 0.015 | 0.020 | -0.025 | 0.055 | 0.471 | 0.934 | 2523 |
| R_superiortemporal_surfavg | -0.011 | 0.027 | -0.064 | 0.042 | 0.685 | 0.934 | 2454 |
| R_supramarginal_surfavg | 0.008 | 0.033 | -0.057 | 0.072 | 0.817 | 0.945 | 2441 |
| R_frontalpole_surfavg | 0.009 | 0.023 | -0.035 | 0.053 | 0.691 | 0.934 | 2566 |
| R_temporalpole_surfavg | -0.034 | 0.020 | -0.073 | 0.005 | 0.089 | 0.636 | 2462 |
| R_transversetemporal_surfavg | -0.038 | 0.031 | -0.099 | 0.023 | 0.221 | 0.836 | 2579 |
| R_insula_surfavg | -0.034 | 0.034 | -0.100 | 0.031 | 0.306 | 0.900 | 2458 |

# Table S14. Association between SA and negative schizotypy corrected for age, and sex

| DKT Regions | Partial R | Std. Err. | 95% CI, lower bound | 95% CI, upper bound | p-value | FDR p-value | Sample size per region |
| --- | --- | --- | --- | --- | --- | --- | --- |
| L_bankssts_surfavg | 0.009 | 0.021 | -0.032 | 0.049 | 0.676 | 0.972 | 2297 |
| L_caudalanteriorcingulate_surfavg | 0.007 | 0.036 | -0.063 | 0.077 | 0.839 | 0.973 | 2446 |
| L_caudalmiddlefrontal_surfavg | -0.008 | 0.020 | -0.048 | 0.031 | 0.687 | 0.972 | 2449 |
| L_cuneus_surfavg | -0.027 | 0.031 | -0.087 | 0.033 | 0.375 | 0.972 | 2322 |
| L_entorhinal_surfavg | -0.026 | 0.034 | -0.092 | 0.040 | 0.444 | 0.972 | 2284 |
| L_fusiform_surfavg | -0.027 | 0.031 | -0.088 | 0.033 | 0.378 | 0.972 | 2414 |
| L_inferiorparietal_surfavg | -0.033 | 0.025 | -0.083 | 0.016 | 0.186 | 0.972 | 2360 |
| L_inferiortemporal_surfavg | 0.011 | 0.026 | -0.039 | 0.061 | 0.672 | 0.972 | 2385 |
| L_isthmuscingulate_surfavg | 0.041 | 0.020 | 0.002 | 0.080 | 0.042 | 0.972 | 2460 |
| L_lateraloccipital_surfavg | -0.031 | 0.020 | -0.071 | 0.009 | 0.131 | 0.972 | 2418 |
| L_lateralorbitofrontal_surfavg | 0.002 | 0.030 | -0.058 | 0.061 | 0.961 | 0.975 | 2434 |
| L_lingual_surfavg | 0.027 | 0.023 | -0.019 | 0.073 | 0.246 | 0.972 | 2401 |
| L_medialorbitofrontal_surfavg | 0.005 | 0.034 | -0.061 | 0.072 | 0.871 | 0.973 | 2418 |
| L_middletemporal_surfavg | 0.009 | 0.021 | -0.032 | 0.050 | 0.675 | 0.972 | 2287 |
| L_parahippocampal_surfavg | -0.020 | 0.020 | -0.059 | 0.020 | 0.324 | 0.972 | 2444 |
| L_paracentral_surfavg | 0.014 | 0.020 | -0.026 | 0.053 | 0.497 | 0.972 | 2457 |
| L_parsopercularis_surfavg | 0.019 | 0.029 | -0.038 | 0.076 | 0.515 | 0.972 | 2434 |
| L_parsorbitalis_surfavg | 0.033 | 0.029 | -0.024 | 0.089 | 0.259 | 0.972 | 2438 |
| L_parstriangularis_surfavg | 0.013 | 0.023 | -0.033 | 0.059 | 0.565 | 0.972 | 2423 |
| L_pericalcarine_surfavg | -0.008 | 0.020 | -0.048 | 0.032 | 0.701 | 0.972 | 2376 |
| L_postcentral_surfavg | 0.023 | 0.023 | -0.021 | 0.067 | 0.306 | 0.972 | 2399 |
| L_posteriorcingulate_surfavg | 0.049 | 0.022 | 0.006 | 0.091 | 0.025 | 0.972 | 2457 |
| L_precentral_surfavg | 0.021 | 0.034 | -0.046 | 0.089 | 0.535 | 0.972 | 2419 |
| L_precuneus_surfavg | -0.012 | 0.021 | -0.053 | 0.029 | 0.565 | 0.972 | 2445 |
| L_rostralanteriorcingulate_surfavg | -0.023 | 0.024 | -0.070 | 0.024 | 0.341 | 0.972 | 2432 |
| L_rostralmiddlefrontal_surfavg | 0.015 | 0.020 | -0.025 | 0.054 | 0.459 | 0.972 | 2439 |
| L_superiorfrontal_surfavg | 0.046 | 0.049 | -0.051 | 0.143 | 0.352 | 0.972 | 2439 |
| L_superiorparietal_surfavg | -0.005 | 0.030 | -0.063 | 0.054 | 0.879 | 0.973 | 2398 |
| L_superiortemporal_surfavg | -0.005 | 0.036 | -0.075 | 0.064 | 0.882 | 0.973 | 2274 |
| L_supramarginal_surfavg | 0.042 | 0.032 | -0.020 | 0.104 | 0.189 | 0.972 | 2307 |
| L_frontalpole_surfavg | -0.028 | 0.030 | -0.086 | 0.030 | 0.348 | 0.972 | 2465 |
| L_temporalpole_surfavg | -0.010 | 0.020 | -0.050 | 0.029 | 0.609 | 0.972 | 2413 |
| L_transversetemporal_surfavg | -0.012 | 0.020 | -0.052 | 0.027 | 0.535 | 0.972 | 2465 |
| L_insula_surfavg | 0.003 | 0.020 | -0.037 | 0.043 | 0.892 | 0.973 | 2392 |
| R_bankssts_surfavg | 0.004 | 0.024 | -0.042 | 0.051 | 0.853 | 0.973 | 2397 |
| R_caudalanteriorcingulate_surfavg | -0.026 | 0.032 | -0.088 | 0.036 | 0.406 | 0.972 | 2456 |
| R_caudalmiddlefrontal_surfavg | -0.008 | 0.020 | -0.048 | 0.031 | 0.681 | 0.972 | 2441 |
| R_cuneus_surfavg | 0.002 | 0.031 | -0.059 | 0.062 | 0.960 | 0.975 | 2347 |
| R_entorhinal_surfavg | -0.032 | 0.034 | -0.099 | 0.035 | 0.354 | 0.972 | 2203 |
| R_fusiform_surfavg | 0.013 | 0.040 | -0.066 | 0.091 | 0.752 | 0.973 | 2421 |
| R_inferiorparietal_surfavg | -0.014 | 0.028 | -0.068 | 0.041 | 0.622 | 0.972 | 2359 |
| R_inferiortemporal_surfavg | -0.021 | 0.034 | -0.086 | 0.045 | 0.540 | 0.972 | 2395 |
| R_isthmuscingulate_surfavg | 0.027 | 0.023 | -0.019 | 0.073 | 0.246 | 0.972 | 2448 |
| R_lateraloccipital_surfavg | -0.019 | 0.020 | -0.059 | 0.021 | 0.354 | 0.972 | 2412 |
| R_lateralorbitofrontal_surfavg | 0.018 | 0.020 | -0.022 | 0.058 | 0.377 | 0.972 | 2410 |
| R_lingual_surfavg | 0.007 | 0.032 | -0.055 | 0.070 | 0.816 | 0.973 | 2405 |
| R_medialorbitofrontal_surfavg | -0.031 | 0.037 | -0.103 | 0.041 | 0.402 | 0.972 | 2397 |
| R_middletemporal_surfavg | 0.012 | 0.027 | -0.040 | 0.064 | 0.656 | 0.972 | 2369 |
| R_parahippocampal_surfavg | -0.007 | 0.021 | -0.049 | 0.035 | 0.741 | 0.973 | 2447 |
| R_paracentral_surfavg | 0.003 | 0.026 | -0.047 | 0.053 | 0.916 | 0.973 | 2454 |
| R_parsopercularis_surfavg | 0.031 | 0.028 | -0.024 | 0.085 | 0.267 | 0.972 | 2411 |
| R_parsorbitalis_surfavg | -0.021 | 0.031 | -0.081 | 0.040 | 0.504 | 0.972 | 2436 |
| R_parstriangularis_surfavg | 0.030 | 0.024 | -0.017 | 0.078 | 0.206 | 0.972 | 2402 |
| R_pericalcarine_surfavg | -0.004 | 0.030 | -0.063 | 0.056 | 0.907 | 0.973 | 2360 |
| R_postcentral_surfavg | 0.001 | 0.020 | -0.038 | 0.041 | 0.943 | 0.975 | 2415 |
| R_posteriorcingulate_surfavg | -0.012 | 0.020 | -0.051 | 0.027 | 0.553 | 0.972 | 2458 |
| R_precentral_surfavg | 0.004 | 0.027 | -0.048 | 0.056 | 0.868 | 0.973 | 2418 |
| R_precuneus_surfavg | 0.023 | 0.021 | -0.019 | 0.065 | 0.286 | 0.972 | 2444 |
| R_rostralanteriorcingulate_surfavg | -0.008 | 0.028 | -0.063 | 0.048 | 0.783 | 0.973 | 2407 |
| R_rostralmiddlefrontal_surfavg | 0.009 | 0.021 | -0.032 | 0.050 | 0.664 | 0.972 | 2417 |
| R_superiorfrontal_surfavg | 0.011 | 0.020 | -0.029 | 0.051 | 0.584 | 0.972 | 2438 |
| R_superiorparietal_surfavg | -0.011 | 0.032 | -0.073 | 0.051 | 0.731 | 0.973 | 2414 |
| R_superiortemporal_surfavg | 0.024 | 0.021 | -0.016 | 0.065 | 0.233 | 0.972 | 2346 |
| R_supramarginal_surfavg | 0.000 | 0.025 | -0.048 | 0.048 | 0.999 | 0.999 | 2333 |
| R_frontalpole_surfavg | 0.015 | 0.029 | -0.042 | 0.072 | 0.609 | 0.972 | 2455 |
| R_temporalpole_surfavg | -0.034 | 0.021 | -0.074 | 0.007 | 0.103 | 0.972 | 2351 |
| R_transversetemporal_surfavg | -0.019 | 0.020 | -0.058 | 0.021 | 0.353 | 0.972 | 2468 |
| R_insula_surfavg | 0.003 | 0.021 | -0.037 | 0.044 | 0.875 | 0.973 | 2359 |

# Table S15. Association between SV and positive schizotypy corrected for age, and sex

| DKT Regions | Partial R | Std. Err. | 95% CI, lower bound | 95% CI, upper bound | p-value | FDR p-value | Sample size per region |
| --- | --- | --- | --- | --- | --- | --- | --- |
| Laccumb | -0.028 | 0.031 | -0.089 | 0.032 | 0.355 | 0.724 | 2626 |
| Lamyg | -0.003 | 0.025 | -0.051 | 0.045 | 0.896 | 0.896 | 2635 |
| Lcaud | 0.032 | 0.019 | -0.006 | 0.070 | 0.103 | 0.724 | 2640 |
| Lhippo | 0.009 | 0.026 | -0.041 | 0.060 | 0.719 | 0.896 | 2633 |
| Lpal | -0.040 | 0.031 | -0.101 | 0.020 | 0.190 | 0.724 | 2427 |
| Lput | -0.016 | 0.020 | -0.054 | 0.022 | 0.407 | 0.724 | 2591 |
| Lthal | -0.011 | 0.030 | -0.070 | 0.048 | 0.713 | 0.896 | 2624 |
| Raccumb | -0.005 | 0.033 | -0.070 | 0.060 | 0.878 | 0.896 | 2624 |
| Ramyg | -0.013 | 0.022 | -0.055 | 0.030 | 0.562 | 0.896 | 2630 |
| Rcaud | 0.019 | 0.019 | -0.019 | 0.057 | 0.331 | 0.724 | 2641 |
| Rhippo | 0.008 | 0.025 | -0.041 | 0.057 | 0.749 | 0.896 | 2642 |
| Rpal | -0.058 | 0.034 | -0.124 | 0.008 | 0.087 | 0.724 | 2627 |
| Rput | -0.016 | 0.019 | -0.055 | 0.022 | 0.396 | 0.724 | 2625 |
| Rthal | 0.009 | 0.032 | -0.054 | 0.072 | 0.785 | 0.896 | 2639 |

# Table S16. Association between SV and negative schizotypy corrected for age, and sex

| DKT Regions | Partial R | Std. Err. | 95% CI, lower bound | 95% CI, upper bound | p-value | FDR p-value | Sample size per region |
| --- | --- | --- | --- | --- | --- | --- | --- |
| Laccumb | -0.031 | 0.025 | -0.080 | 0.017 | 0.202 | 0.429 | 2658 |
| Lamyg | 0.026 | 0.022 | -0.017 | 0.068 | 0.231 | 0.437 | 2667 |
| Lcaud | 0.030 | 0.022 | -0.012 | 0.073 | 0.164 | 0.429 | 2675 |
| Lhippo | 0.034 | 0.021 | -0.008 | 0.076 | 0.113 | 0.429 | 2669 |
| Lpal | -0.041 | 0.023 | -0.086 | 0.003 | 0.070 | 0.429 | 2458 |
| Lput | -0.017 | 0.031 | -0.078 | 0.043 | 0.573 | 0.749 | 2629 |
| Lthal | -0.009 | 0.032 | -0.072 | 0.054 | 0.782 | 0.831 | 2659 |
| Raccumb | 0.001 | 0.032 | -0.061 | 0.063 | 0.978 | 0.978 | 2657 |
| Ramyg | 0.008 | 0.025 | -0.041 | 0.057 | 0.759 | 0.831 | 2662 |
| Rcaud | 0.013 | 0.022 | -0.030 | 0.056 | 0.542 | 0.749 | 2677 |
| Rhippo | 0.029 | 0.022 | -0.014 | 0.071 | 0.189 | 0.429 | 2676 |
| Rpal | -0.034 | 0.019 | -0.072 | 0.004 | 0.081 | 0.429 | 2663 |
| Rput | -0.010 | 0.027 | -0.064 | 0.044 | 0.714 | 0.831 | 2662 |
| Rthal | -0.028 | 0.039 | -0.106 | 0.049 | 0.472 | 0.729 | 2678 |

# Fig S1. Associations between CT profiles of positive and negative schizotypy with those from CHR stages and schizophrenia

# Molecular, connectivity and neurotransmitter contributions to cortical morphology of schizotypy dimensions

We examined how cortical profiles in positive and negative schizotypy are informed by three different sets of molecular (or microarchitectural), normative structural connectivity, and specific neurotransmitter measures. All molecular, structural connectivity, and neurotransmitter maps provide regional values for each of the 68 DKT regions and were derived from two recent publication [1, 2]. Details on all molecular, connectivity, and neurotransmitter maps can be found in the supplement as well as in the publications from Hansen and colleagues [1, 2]. Data can be obtained here: <https://github.com/netneurolab/hansen_crossdisorder_vulnerability.git>, <https://github.com/netneurolab/hansen_receptors.git>.

**Molecular predictors.** Following Hansen and colleagues [1] we used a total of seven local molecular predictors in the multilinear models to examine the influence that local molecular attributes have on cortical morphology of positive and negative schizotypy.

**Gene expression gradient.** The first principal component of gene expression (“gene gradient”) was used to represent the variation in gene expression levels across the left cortex. Gene expression data of in total 11,560 genes derived from the Allen Human Brain Atlas as described in Hawrylycz[3] and processed by *abagen*, an open-source Python toolbox [4].

**Receptor gradient.** The first principal component of receptor density (“receptor gradient”) was used to represent the variation in receptor densities across the cortex. Receptor densities were estimated using positron emission tomography (PET) tracer studies in healthy volunteers, for a total of 18 receptors and transporters, across 9 neurotransmitter systems. For details on the included receptors and transporters see [2].

**Glycolytic index.** This brain map provides glycolytic index values defined as the residual after fitting glucose metabolism to oxygen metabolism in a linear regression model. Larger values indicate more aerobic glycolysis. Original data were collected, calculated, and made available by Vaishnavi et al[5].

**Glucose metabolism**. The glucose metabolism cortical map is based on [^18^F] -labelled fluorodeoxyglucose (FDG) PET scans in 33 healthy adults (19 female, mean age 25.4 ± 2.6 years) as described in detail in Vaishnavi et al. [5].

**Synapse density.** The synapse density cortical maps is based on PET scans in 76 healthy adults (45 males, 48.9 ± 18.4 years of age) using [^11^C]UCB-J, a PET tracer that binds to the synaptic vesicle glycoprotein 2A (SV2A).

**Myelination.** The cortical myelination map is based on T1w/T2w ratios from healthy individuals (n=417, age range 22–37 years, 193 males) of the Human Connectome Project (HCP, S1200 release)[6, 7].

**Connectivity predictors.** Following [1], a total of nine global connectome predictors computed on weighted structural connectome were used in the multilinear model to examine their influence on cortical morphology of positive and negative schizotypy. Structural group-level connectome data are based on a healthy cohort (16 females, 25.3 ± 4.9) collected at the Department of Radiology, University Hospital Center and University of Lausanne. Structural connectivity estimation and group-consensus structural network generation are described in detail in Hansen et al. [1]. All connectivity measures were computed using the Python-equivalent of the Brain Connectivity Toolbox, bctpy. Connectivity predictors included: strength = sum of weighted connections; betweenness = fraction of all shortest paths traversing region i; closeness = mean shortest path length between region i and all other regions; Euclidean distance = mean Euclidean distance between region i and all other regions; participation coefficient = diversity of connections from region i to the seven Yeo-Krienen resting-state networks [8]; clustering = fraction of triangles including region i; mean first passage time = average time for a random walker to travel from region i to any other region.

**Neurotransmitter predictors.** Neurotransmitter predictors used in the multilinear models included 18 cortical receptor/transporter maps derived from PET tracer studies [2]. These include dopamine (D1, D2, DAT), norepinephrine (NET), serotonin (5-HT1A, 5-HT1B, 5-HT2A, 5-HT4, 5-HT6, 5-HTT), acetylcholine (α4β2, M1, VAChT), glutamate (mGluR5), GABA (GABA_A_), histamine (H3), cannabinoid (CB1), and opioid (MOR). Data collection and processing of all PET maps into 68 cortical DKT regions is described in detail in Hansen et al [2].

# Fig S2. Normative functional degree centrality derived from the HCP sample (n=207)

# Fig S3. Normative structural degree centrality derived from the HCP sample (n=207)

# Table S17. Functional epicenters of associations between CT and positive schizotypy

| DKT regions | Functional epicenter r-values | pspin |
| --- | --- | --- |
| L_inferiortemporal_thickavg | -0.541680148 | 0.001 |
| R_fusiform_thickavg | -0.512515271 | 0.0015 |
| L_precentral_thickavg | -0.502460361 | 0.0015 |
| R_paracentral_thickavg | -0.48829117 | 0.002 |
| L_superiorparietal_thickavg | -0.468902802 | 0.0015 |
| L_supramarginal_thickavg | -0.466722129 | 0.003 |
| L_paracentral_thickavg | -0.465377588 | 0.0025 |
| R_supramarginal_thickavg | -0.465268837 | 0.0025 |
| L_transversetemporal_thickavg | -0.463268157 | 0.001 |
| R_precentral_thickavg | -0.458660906 | 0.003 |
| R_insula_thickavg | -0.453906665 | 0.0025 |
| L_lateraloccipital_thickavg | -0.450966614 | 0 |
| L_parstriangularis_thickavg | -0.449613694 | 0.001 |
| L_fusiform_thickavg | -0.447597364 | 0.0025 |
| R_superiorparietal_thickavg | -0.444430332 | 0.0035 |
| R_postcentral_thickavg | -0.442507552 | 0.0025 |
| R_bankssts_thickavg | -0.439967713 | 0.0025 |
| L_insula_thickavg | -0.43082653 | 0.0055 |
| R_parsopercularis_thickavg | -0.43029572 | 0.0025 |
| L_postcentral_thickavg | -0.424835738 | 0.005 |
| R_pericalcarine_thickavg | -0.416999046 | 0.0065 |
| R_cuneus_thickavg | -0.413254388 | 0.0095 |
| L_caudalanteriorcingulate_thickavg | -0.410612027 | 0.0095 |
| L_bankssts_thickavg | -0.401772067 | 0.0075 |
| R_lingual_thickavg | -0.400782585 | 0.0185 |
| L_superiortemporal_thickavg | -0.39896963 | 0.0085 |
| R_inferiortemporal_thickavg | -0.391763266 | 0.0155 |
| L_parsopercularis_thickavg | -0.388610204 | 0.01 |
| L_rostralmiddlefrontal_thickavg | -0.382626214 | 0.0065 |
| R_superiortemporal_thickavg | -0.380983749 | 0.016 |
| L_cuneus_thickavg | -0.379974987 | 0.01 |
| L_lingual_thickavg | -0.379200675 | 0.0185 |
| R_transversetemporal_thickavg | -0.372581011 | 0.025 |
| L_pericalcarine_thickavg | -0.352034631 | 0.0215 |
| R_caudalanteriorcingulate_thickavg | -0.337108828 | 0.0265 |
| L_posteriorcingulate_thickavg | -0.329816534 | 0.0255 |
| R_posteriorcingulate_thickavg | -0.32437978 | 0.0285 |
| L_superiorfrontal_thickavg | -0.322737804 | 0.0305 |
| L_caudalmiddlefrontal_thickavg | -0.315727326 | 0.0155 |
| L_entorhinal_thickavg | -0.31329959 | 0.036 |
| R_lateraloccipital_thickavg | -0.306338903 | 0.0495 |
| R_parstriangularis_thickavg | -0.287850241 | 0.041 |
| L_lateralorbitofrontal_thickavg | -0.277072976 | 0.074 |
| R_lateralorbitofrontal_thickavg | -0.272083636 | 0.029 |
| R_rostralmiddlefrontal_thickavg | -0.256911327 | 0.0405 |
| R_entorhinal_thickavg | -0.255044799 | 0.05 |
| R_superiorfrontal_thickavg | -0.239138313 | 0.0735 |
| R_caudalmiddlefrontal_thickavg | -0.224098748 | 0.046 |
| L_middletemporal_thickavg | -0.222989625 | 0.056 |
| L_parahippocampal_thickavg | -0.216029004 | 0.121 |
| R_inferiorparietal_thickavg | -0.199302834 | 0.084 |
| R_middletemporal_thickavg | -0.190080766 | 0.07 |
| L_inferiorparietal_thickavg | -0.186544165 | 0.103 |
| R_parahippocampal_thickavg | -0.177121865 | 0.219 |
| L_parsorbitalis_thickavg | -0.160661335 | 0.212 |
| R_precuneus_thickavg | -0.157037594 | 0.2175 |
| L_precuneus_thickavg | -0.140488629 | 0.228 |
| L_temporalpole_thickavg | -0.105385341 | 0.305 |
| R_parsorbitalis_thickavg | -0.091513058 | 0.2655 |
| R_isthmuscingulate_thickavg | -0.046090321 | 0.4165 |
| L_isthmuscingulate_thickavg | 0.011160005 | 0.4335 |
| L_medialorbitofrontal_thickavg | 0.021895841 | 0.3865 |
| R_frontalpole_thickavg | 0.086776598 | 0.244 |
| L_frontalpole_thickavg | 0.096296975 | 0.1715 |
| R_temporalpole_thickavg | 0.129917582 | 0.209 |
| L_rostralanteriorcingulate_thickavg | 0.175427439 | 0.108 |
| R_rostralanteriorcingulate_thickavg | 0.23721669 | 0.0615 |
| R_medialorbitofrontal_thickavg | 0.258349688 | 0.032 |

# Table S18. Structural epicenters of associations between CT and positive schizotypy

| DKT regions | Structural epicenter r-values | pspin |
| --- | --- | --- |
| L_precentral_thickavg | -0.450527433 | 0.01 |
| L_superiorparietal_thickavg | -0.387073376 | 0.019 |
| L_postcentral_thickavg | -0.346522677 | 0.0525 |
| L_supramarginal_thickavg | -0.332381842 | 0.0385 |
| R_postcentral_thickavg | -0.296875232 | 0.0515 |
| R_paracentral_thickavg | -0.28088864 | 0.087 |
| R_caudalmiddlefrontal_thickavg | -0.280172167 | 0.032 |
| R_superiorparietal_thickavg | -0.270674693 | 0.0315 |
| L_caudalmiddlefrontal_thickavg | -0.26417868 | 0.107 |
| L_paracentral_thickavg | -0.25620386 | 0.1445 |
| R_precentral_thickavg | -0.245620138 | 0.0835 |
| L_inferiorparietal_thickavg | -0.242205114 | 0.08 |
| L_cuneus_thickavg | -0.234810227 | 0.1475 |
| L_middletemporal_thickavg | -0.232945211 | 0.0865 |
| L_parsopercularis_thickavg | -0.228817022 | 0.104 |
| R_parsopercularis_thickavg | -0.219468843 | 0.026 |
| R_pericalcarine_thickavg | -0.213011237 | 0.126 |
| L_superiortemporal_thickavg | -0.200442324 | 0.1595 |
| L_parstriangularis_thickavg | -0.183534435 | 0.17 |
| R_cuneus_thickavg | -0.182259429 | 0.1835 |
| L_fusiform_thickavg | -0.174608545 | 0.2235 |
| R_supramarginal_thickavg | -0.161567074 | 0.085 |
| L_transversetemporal_thickavg | -0.156825374 | 0.197 |
| L_pericalcarine_thickavg | -0.153830126 | 0.268 |
| L_rostralmiddlefrontal_thickavg | -0.142202204 | 0.2555 |
| R_inferiorparietal_thickavg | -0.140757458 | 0.0385 |
| L_inferiortemporal_thickavg | -0.139842846 | 0.233 |
| L_parahippocampal_thickavg | -0.136357659 | 0.308 |
| L_precuneus_thickavg | -0.134178638 | 0.308 |
| L_lateraloccipital_thickavg | -0.129769313 | 0.3235 |
| L_insula_thickavg | -0.113380386 | 0.3345 |
| L_isthmuscingulate_thickavg | -0.079776739 | 0.396 |
| L_parsorbitalis_thickavg | -0.058150885 | 0.455 |
| R_middletemporal_thickavg | -0.056731328 | 0.117 |
| L_bankssts_thickavg | -0.054444487 | 0.448 |
| R_posteriorcingulate_thickavg | -0.053780861 | 0.413 |
| R_fusiform_thickavg | -0.051653246 | 0.2025 |
| R_parstriangularis_thickavg | -0.043190004 | 0.255 |
| L_lingual_thickavg | -0.038636232 | 0.4695 |
| R_lingual_thickavg | -0.026244175 | 0.3805 |
| R_lateraloccipital_thickavg | -0.023249738 | 0.3095 |
| R_precuneus_thickavg | -0.016512143 | 0.3905 |
| R_superiortemporal_thickavg | -0.013499584 | 0.323 |
| R_isthmuscingulate_thickavg | -0.007934888 | 0.4175 |
| R_insula_thickavg | 0.021073885 | 0.8015 |
| R_rostralmiddlefrontal_thickavg | 0.030814987 | 0.5095 |
| R_transversetemporal_thickavg | 0.032547412 | 0.4865 |
| R_frontalpole_thickavg | 0.058154123 | 0.434 |
| L_superiorfrontal_thickavg | 0.064981736 | 0.383 |
| R_inferiortemporal_thickavg | 0.065164281 | 0.4465 |
| L_temporalpole_thickavg | 0.099028243 | 0.205 |
| R_bankssts_thickavg | 0.101564115 | 0.3085 |
| R_parsorbitalis_thickavg | 0.102204491 | 0.336 |
| R_entorhinal_thickavg | 0.102875849 | 0.354 |
| L_entorhinal_thickavg | 0.116996487 | 0.1665 |
| R_parahippocampal_thickavg | 0.121845215 | 0.3505 |
| R_superiorfrontal_thickavg | 0.126388892 | 0.321 |
| L_posteriorcingulate_thickavg | 0.153332731 | 0.2245 |
| L_frontalpole_thickavg | 0.185829054 | 0.1745 |
| L_caudalanteriorcingulate_thickavg | 0.199543337 | 0.096 |
| L_lateralorbitofrontal_thickavg | 0.210287497 | 0.078 |
| R_temporalpole_thickavg | 0.260177451 | 0.066 |
| R_rostralanteriorcingulate_thickavg | 0.274163057 | 0.072 |
| L_rostralanteriorcingulate_thickavg | 0.284256954 | 0.0455 |
| R_caudalanteriorcingulate_thickavg | 0.290121173 | 0.0655 |
| L_medialorbitofrontal_thickavg | 0.315712575 | 0.0295 |
| R_lateralorbitofrontal_thickavg | 0.324852123 | 0.0425 |
| R_medialorbitofrontal_thickavg | 0.352655488 | 0.025 |

# Table S19. Functional epicenters of associations between CT and negative schizotypy

| DKT regions | Functional epicenter r-values | pspin |
| --- | --- | --- |
| R_fusiform_thickavg | -0.486010279 | 0.003 |
| R_superiortemporal_thickavg | -0.441189947 | 0.0095 |
| L_bankssts_thickavg | -0.439478655 | 0.0095 |
| L_superiortemporal_thickavg | -0.428526131 | 0.018 |
| L_inferiortemporal_thickavg | -0.4282116 | 0.0095 |
| R_superiorparietal_thickavg | -0.424477627 | 0.0085 |
| L_fusiform_thickavg | -0.422526419 | 0.015 |
| L_entorhinal_thickavg | -0.416708471 | 0.0225 |
| L_postcentral_thickavg | -0.415400515 | 0.004 |
| R_paracentral_thickavg | -0.414600402 | 0.015 |
| L_parahippocampal_thickavg | -0.404285021 | 0.008 |
| R_precentral_thickavg | -0.400258903 | 0.0105 |
| L_transversetemporal_thickavg | -0.398740616 | 0.0175 |
| L_superiorparietal_thickavg | -0.39756996 | 0.0085 |
| L_insula_thickavg | -0.387598935 | 0.025 |
| R_inferiortemporal_thickavg | -0.373901367 | 0.0245 |
| R_lateraloccipital_thickavg | -0.371486939 | 0.0105 |
| R_pericalcarine_thickavg | -0.369244987 | 0.0125 |
| L_precentral_thickavg | -0.367821631 | 0.0305 |
| R_lingual_thickavg | -0.361181989 | 0.049 |
| L_paracentral_thickavg | -0.356513044 | 0.041 |
| R_parahippocampal_thickavg | -0.351400486 | 0.036 |
| R_supramarginal_thickavg | -0.348486851 | 0.0265 |
| L_supramarginal_thickavg | -0.347902378 | 0.039 |
| L_lateraloccipital_thickavg | -0.342756874 | 0.0145 |
| R_transversetemporal_thickavg | -0.335401738 | 0.0565 |
| R_posteriorcingulate_thickavg | -0.333242032 | 0.027 |
| L_cuneus_thickavg | -0.328429022 | 0.036 |
| R_postcentral_thickavg | -0.32662095 | 0.0545 |
| L_posteriorcingulate_thickavg | -0.326448996 | 0.0545 |
| R_entorhinal_thickavg | -0.324518811 | 0.013 |
| R_cuneus_thickavg | -0.316737326 | 0.057 |
| L_superiorfrontal_thickavg | -0.316328742 | 0.0785 |
| R_bankssts_thickavg | -0.31575878 | 0.0725 |
| L_caudalmiddlefrontal_thickavg | -0.303260063 | 0.0525 |
| R_insula_thickavg | -0.295932794 | 0.0765 |
| R_superiorfrontal_thickavg | -0.292868364 | 0.048 |
| R_caudalanteriorcingulate_thickavg | -0.288039176 | 0.0545 |
| L_parstriangularis_thickavg | -0.275081808 | 0.1445 |
| L_precuneus_thickavg | -0.269198416 | 0.0705 |
| L_lingual_thickavg | -0.258816837 | 0.104 |
| L_pericalcarine_thickavg | -0.258170425 | 0.079 |
| L_caudalanteriorcingulate_thickavg | -0.252185783 | 0.105 |
| L_temporalpole_thickavg | -0.250454461 | 0.1015 |
| R_caudalmiddlefrontal_thickavg | -0.247540655 | 0.032 |
| L_rostralmiddlefrontal_thickavg | -0.246358468 | 0.1595 |
| L_parsopercularis_thickavg | -0.244155873 | 0.19 |
| L_inferiorparietal_thickavg | -0.243111634 | 0.0795 |
| R_precuneus_thickavg | -0.242577544 | 0.12 |
| R_inferiorparietal_thickavg | -0.219461536 | 0.048 |
| R_parsopercularis_thickavg | -0.216106526 | 0.1065 |
| L_lateralorbitofrontal_thickavg | -0.209305933 | 0.2295 |
| R_parstriangularis_thickavg | -0.207308696 | 0.123 |
| L_middletemporal_thickavg | -0.180481979 | 0.181 |
| R_middletemporal_thickavg | -0.134239674 | 0.1565 |
| L_parsorbitalis_thickavg | -0.129350392 | 0.4135 |
| R_isthmuscingulate_thickavg | -0.122828013 | 0.3025 |
| L_isthmuscingulate_thickavg | -0.113145869 | 0.341 |
| R_temporalpole_thickavg | -0.093699235 | 0.2425 |
| R_rostralmiddlefrontal_thickavg | -0.06805229 | 0.281 |
| L_medialorbitofrontal_thickavg | -0.059581721 | 0.457 |
| R_lateralorbitofrontal_thickavg | -0.052411493 | 0.328 |
| L_frontalpole_thickavg | -0.040604691 | 0.5905 |
| R_medialorbitofrontal_thickavg | -0.018746628 | 0.4835 |
| R_parsorbitalis_thickavg | -0.005409198 | 0.4565 |
| L_rostralanteriorcingulate_thickavg | 0.015941545 | 0.4 |
| R_frontalpole_thickavg | 0.044061744 | 0.307 |
| R_rostralanteriorcingulate_thickavg | 0.121252941 | 0.239 |

# Table S20. Structural epicenters of associations between CT and negative schizotypy

| DKT regions | Structural epicenter r-values | pspin |
| --- | --- | --- |
| L_superiorparietal_thickavg | -0.456381671 | 0.0185 |
| R_superiorparietal_thickavg | -0.362083303 | 0.0015 |
| L_inferiorparietal_thickavg | -0.353785336 | 0.0255 |
| L_fusiform_thickavg | -0.342905438 | 0.0435 |
| L_parahippocampal_thickavg | -0.3276488 | 0.067 |
| L_superiortemporal_thickavg | -0.30776082 | 0.1145 |
| L_pericalcarine_thickavg | -0.299329052 | 0.0945 |
| L_inferiortemporal_thickavg | -0.2860747 | 0.0775 |
| L_middletemporal_thickavg | -0.272028989 | 0.178 |
| R_pericalcarine_thickavg | -0.266706404 | 0.02 |
| L_postcentral_thickavg | -0.263043115 | 0.1235 |
| L_cuneus_thickavg | -0.260733469 | 0.1235 |
| L_lingual_thickavg | -0.25432104 | 0.191 |
| L_supramarginal_thickavg | -0.235610147 | 0.2695 |
| L_bankssts_thickavg | -0.235353145 | 0.107 |
| R_cuneus_thickavg | -0.23356289 | 0.0735 |
| L_precuneus_thickavg | -0.226560308 | 0.2475 |
| L_lateraloccipital_thickavg | -0.202791913 | 0.286 |
| L_isthmuscingulate_thickavg | -0.198459146 | 0.293 |
| L_entorhinal_thickavg | -0.197517862 | 0.284 |
| L_paracentral_thickavg | -0.159553304 | 0.2885 |
| R_lingual_thickavg | -0.157918027 | 0.085 |
| R_supramarginal_thickavg | -0.142391894 | 0.08 |
| R_fusiform_thickavg | -0.138259598 | 0.023 |
| L_precentral_thickavg | -0.13786686 | 0.445 |
| L_temporalpole_thickavg | -0.136628108 | 0.448 |
| L_insula_thickavg | -0.132741897 | 0.8585 |
| R_paracentral_thickavg | -0.132491884 | 0.2995 |
| R_postcentral_thickavg | -0.121645732 | 0.24 |
| L_caudalmiddlefrontal_thickavg | -0.092612607 | 0.5015 |
| L_parstriangularis_thickavg | -0.084343369 | 0.567 |
| L_parsopercularis_thickavg | -0.083138389 | 0.637 |
| L_transversetemporal_thickavg | -0.081260884 | 0.5515 |
| R_superiortemporal_thickavg | -0.07625479 | 0.0855 |
| R_lateraloccipital_thickavg | -0.055094515 | 0.143 |
| R_posteriorcingulate_thickavg | -0.051823428 | 0.4055 |
| L_parsorbitalis_thickavg | -0.030600467 | 0.772 |
| R_inferiortemporal_thickavg | -0.018873909 | 0.205 |
| R_precuneus_thickavg | -0.01315417 | 0.318 |
| R_inferiorparietal_thickavg | -0.010780333 | 0.1185 |
| R_bankssts_thickavg | -0.005790831 | 0.3185 |
| R_temporalpole_thickavg | -0.001652915 | 0.2665 |
| R_entorhinal_thickavg | -0.00095885 | 0.1965 |
| R_transversetemporal_thickavg | 0.003630264 | 0.6545 |
| R_precentral_thickavg | 0.013252736 | 0.5465 |
| R_middletemporal_thickavg | 0.016668482 | 0.8665 |
| L_posteriorcingulate_thickavg | 0.033910472 | 0.3815 |
| R_caudalmiddlefrontal_thickavg | 0.03477334 | 0.499 |
| R_parahippocampal_thickavg | 0.040156936 | 0.71 |
| R_isthmuscingulate_thickavg | 0.053382429 | 0.531 |
| R_parstriangularis_thickavg | 0.087852296 | 0.58 |
| R_parsorbitalis_thickavg | 0.143779998 | 0.4665 |
| L_rostralmiddlefrontal_thickavg | 0.169802347 | 0.0705 |
| L_caudalanteriorcingulate_thickavg | 0.204117461 | 0.0685 |
| R_parsopercularis_thickavg | 0.208877256 | 0.3145 |
| L_lateralorbitofrontal_thickavg | 0.22632278 | 0.034 |
| L_superiorfrontal_thickavg | 0.274993147 | 0.0715 |
| R_superiorfrontal_thickavg | 0.27499686 | 0.19 |
| R_caudalanteriorcingulate_thickavg | 0.286734894 | 0.132 |
| R_rostralmiddlefrontal_thickavg | 0.306496755 | 0.0705 |
| L_rostralanteriorcingulate_thickavg | 0.321708508 | 0.0175 |
| R_frontalpole_thickavg | 0.340200895 | 0.046 |
| L_medialorbitofrontal_thickavg | 0.346424602 | 0.017 |
| R_insula_thickavg | 0.365467806 | 0.029 |
| R_rostralanteriorcingulate_thickavg | 0.376647635 | 0.0365 |
| L_frontalpole_thickavg | 0.39228198 | 0.0175 |
| R_medialorbitofrontal_thickavg | 0.416714908 | 0.027 |
| R_lateralorbitofrontal_thickavg | 0.50482831 | 0.006 |

# Comparison epicenter analysis with spin-permutation null model and network-rewiring null model

In the present study, our primary statistical framework for evaluating epicenter significance relied on spin-permutation null models, which preserve the spatial autocorrelation of cortical maps. This is essential because, like any cortical thickness map, the schizotypy-related cortical effect-size maps exhibit strong spatial autocorrelation, and failing to account for this structure can inflate false positives. Spin-based nulls are widely used in epicenter and hub-vulnerability analyses [1, 9–12] and have been shown to provide robust statistical control across parcellation resolutions [13]. In addition, to spatial auto-correlation preserving spin tests, we also implemented network-rewiring null model which preserves network features such as degree and distance relationships of the underlying connectome matrix [1, 14, 15]. These null models test the complementary hypothesis that the observed epicenter effects cannot be explained by regional variations in node degree or by spatially biased connectivity patterns [1, 16]. Following Chopra et al. [16], we generated **1,000 rewired structural and functional connectomes** using **10 distance bins and 10,000 edge swaps.** The lower number of edge swaps (10,000 vs. 50,000 in Chopra et al. [16]) reflects the smaller size of the DKT-68 connectome (68×68 vs. 332×332 [16]). In rewiring-based null models, the number of required swaps scales with network size, and 10,000 swaps provides sufficient randomization for a 68×68 matrix while preserving degree and distance-bin constraints.

Across both schizotypy dimensions, the network-rewiring null replicated the spatial patterns obtained with spin permutation tests. Specifically, the rewiring null reproduced (i) sensorimotor–associative epicenters for schizotypy-related CT reductions and (ii) more circumscribed mOFC/vmPFC epicenters for schizotypy-related CT increases (Fig. S4).

The strong concordance between the two null frameworks (spin-test vs network-rewiring) was confirmed by robust correlations between their regional p-value maps (positive schizotypy: *r* > 0.8 for both functional and structural epicenters; negative schizotypy: *r* > 0.8 for functional and *r* > 0.6 for structural epicenters). These results indicate that our epicenter findings are not dependent on the choice of null model and are robust to both spatial-structure-preserving and network-topology-preserving null constraints.

# Fig S4. Epicenters derived from spin-permutation and network-rewiring null model

# Funding and Acknowledgements

| FOR 2107-MR | This work was funded by the German Research Foundation (DFG grants FOR2107 KI588/14-1, and KI588/14-2, and KI588/20-1, KI588/22-1 to Tilo Kircher, Marburg, Germany). Biosamples and corresponding data were sampled, processed and stored in the Marburg Biobank CBBMR. |
| --- | --- |
| FOR2107-MS | This work was funded by the German Research Foundation (DFG, grant FOR2107 DA1151/5-1, DA1151/5-2, DA1151/9-1, DA1151/10-1, DA1151/11-1 to UD) and the Interdisciplinary Center for Clinical Research (IZKF) of the medical faculty of M√ºnster (grant Dan3/022/22 to UD). |
| Bonn1, Bonn2, Bonn3 | DFG Et 31/2-1 |
| FOR2107-MR, Jena | DFG (NE2254/1-2, NE2254/2-1, NE2254/3-1, NE2254/4-1) |
| IGP and ASRB | NHMRC APP630471; APP1051672; APP1081603 |
| FIDMAG | AGAUR, Generalitat de Catalunya. CIBERSAM ISCIII |
| Zurich 2 | Collegium Heveticum |
| ENIGMA SZ WG | This work was in part supported by the National Institute of Mental Health of the National Institutes of Health under award numbers R01MH121246 and R01MH1345261. |
| Paris | ANR MNP VIP, ANR Labex BioPsy |
| Geneva | Swiss National Science Foundation grant number 159440 |

# References

1.	Hansen JY, Shafiei G, Vogel JW, Smart K, Bearden CE, Hoogman M, et al. Local molecular and global connectomic contributions to cross-disorder cortical abnormalities. Nat Commun. 2022;13:4682.

2.	Hansen JY, Shafiei G, Markello RD, Smart K, Cox SML, Nørgaard M, et al. Mapping neurotransmitter systems to the structural and functional organization of the human neocortex. Nat Neurosci. 2022;25:1569–1581.

3.	Hawrylycz MJ, Lein ES, Guillozet-Bongaarts AL, Shen EH, Ng L, Miller JA, et al. An anatomically comprehensive atlas of the adult human brain transcriptome. Nature. 2012;489:391–399.

4.	Markello R, Shafiei G, Zheng Y-Q, Mišić B. abagen: A toolbox for the Allen Brain Atlas genetics data. 2020.

5.	Vaishnavi SN, Vlassenko AG, Rundle MM, Snyder AZ, Mintun MA, Raichle ME. Regional aerobic glycolysis in the human brain. Proc Natl Acad Sci U S A. 2010;107:17757–17762.

6.	Van Essen DC, Smith SM, Barch DM, Behrens TEJ, Yacoub E, Ugurbil K. The WU-Minn Human Connectome Project: An Overview. Neuroimage. 2013;80:62–79.

7.	Glasser MF, Sotiropoulos SN, Wilson JA, Coalson TS, Fischl B, Andersson JL, et al. The minimal preprocessing pipelines for the Human Connectome Project. Neuroimage. 2013;80:105–124.

8.	Yeo BTT, Krienen FM, Sepulcre J, Sabuncu MR, Lashkari D, Hollinshead M, et al. The organization of the human cerebral cortex estimated by intrinsic functional connectivity. J Neurophysiol. 2011;106:1125–1165.

9.	Larivière S, Rodríguez-Cruces R, Royer J, Caligiuri ME, Gambardella A, Concha L, et al. Network-based atrophy modeling in the common epilepsies: A worldwide ENIGMA study. Science Advances. 2020;6:eabc6457.

10.	Ngo A, Liu L, Larivière S, Kebets V, Fett S, Weber CF, et al. Associations between epilepsy-related polygenic risk and brain morphology in childhood. Brain. 2025:awaf259.

11.	Georgiadis F, Larivière S, Glahn D, Hong LE, Kochunov P, Mowry B, et al. Connectome architecture shapes large-scale cortical alterations in schizophrenia: a worldwide ENIGMA study. Mol Psychiatry. 2024:1–13.

12.	Hettwer MD, Larivière S, Park BY, van den Heuvel OA, Schmaal L, Andreassen OA, et al. Coordinated cortical thickness alterations across six neurodevelopmental and psychiatric disorders. Nat Commun. 2022;13:6851.

13.	Markello RD, Misic B. Comparing spatially-constrained null models for parcellated brain maps. Neuroscience; 2020.

14.	Gollo LL, Roberts JA, Cropley VL, Di Biase MA, Pantelis C, Zalesky A, et al. Fragility and volatility of structural hubs in the human connectome. Nat Neurosci. 2018;21:1107–1116.

15.	Betzel RF, Bassett DS. Specificity and robustness of long-distance connections in weighted, interareal connectomes. Proc Natl Acad Sci U S A. 2018;115:E4880–E4889.

16.	Chopra S, Segal A, Oldham S, Holmes A, Sabaroedin K, Orchard ER, et al. Network-Based Spreading of Gray Matter Changes Across Different Stages of Psychosis. JAMA Psychiatry. 2023;80:1246–1257.
